# Natural phenolic compounds as biofilm inhibitors of multidrug-resistant *Escherichia coli* – the role of similar biological processes despite structural diversity

**DOI:** 10.3389/fmicb.2023.1232039

**Published:** 2023-09-04

**Authors:** David Buchmann, Michael Schwabe, Romano Weiss, Andreas W. Kuss, Katharina Schaufler, Rabea Schlüter, Stefan Rödiger, Sebastian Guenther, Nadin Schultze

**Affiliations:** ^1^Pharmaceutical Biology, Institute of Pharmacy, University of Greifswald, Greifswald, Germany; ^2^Pharmaceutical Microbiology, Institute of Pharmacy, University of Greifswald, Greifswald, Germany; ^3^Institute of Biotechnology, Faculty Environment and Natural Sciences, Brandenburg University of Technology Cottbus-Senftenberg, Cottbus, Germany; ^4^Department of Functional Genomics, Interfaculty Institute of Genetics and Functional Genomics, University of Greifswald, Greifswald, Germany; ^5^Institute of Infection Medicine, Christian-Albrecht University Kiel and University Medical Center Schleswig-Holstein, Kiel, Germany; ^6^Imaging Center of the Department of Biology, University of Greifswald, Greifswald, Germany

**Keywords:** phenolic compounds, biofilm inhibitor, *Escherichia coli*, transcriptome, RNA-Seq, motility, metabolic processes, arginine biosynthesis

## Abstract

Multidrug-resistant gram-negative pathogens such as *Escherichia coli* have become increasingly difficult to treat and therefore alternative treatment options are needed. Targeting virulence factors like biofilm formation could be one such option. Inhibition of biofilm-related structures like curli and cellulose formation in *E. coli* has been shown for different phenolic natural compounds like epigallocatechin gallate. This study demonstrates this effect for other structurally unrelated phenolics, namely octyl gallate, scutellarein and wedelolactone. To verify whether these structurally different compounds influence identical pathways of biofilm formation in *E. coli* a broad comparative RNA-sequencing approach was chosen with additional RT-qPCR to gain initial insights into the pathways affected at the transcriptomic level. Bioinformatical analysis of the RNA-Seq data was performed using DESeq2, BioCyc and KEGG Mapper. The comparative bioinformatics analysis on the pathways revealed that, irrespective of their structure, all compounds mainly influenced similar biological processes. These pathways included bacterial motility, chemotaxis, biofilm formation as well as metabolic processes like arginine biosynthesis and tricarboxylic acid cycle. Overall, this work provides the first insights into the potential mechanisms of action of novel phenolic biofilm inhibitors and highlights the complex regulatory processes of biofilm formation in *E. coli*.

## 1. Introduction

There are countless natural compounds and more than a quarter of all medicines approved by the Food and Drug Administration (FDA) or the European Medical Agency (EMA) have natural models or are plant-based (Thomford et al., 2018). This is also true for those used to treat infections. Numerous studies have reported antimicrobial properties of extracts and natural products (Cowan, 1999; van Vuuren and Viljoen, 2011; Chandra et al., 2017; Guglielmi et al., 2020), which are important, considering the urgent need for new antimicrobial agents, especially for use against multidrug-resistant gram-negatives (MDR-GN). These compounds are valuable resources for new drug development. According to the WHO Global Priority List 2017 (World Health Organization, 2017) MDR-GN are of particular importance with regard to the development of new drugs for the fight against the global threat caused by bacterial resistance (World Health Organization, 2019).

In recent years, in addition to the search for direct bacteriostatic or bactericidal substances, research has focused on specific virulence targets of pathogens (Wu et al., 2008; Manner et al., 2013; Terlizzi et al., 2017). The main advantage here lies in the reduced selection pressure on the overall microbiota, as active substances specifically attenuate the virulence of the addressed pathogens (Clatworthy et al., 2007; Sivick and Mobley, 2010). As about 65% of bacterial diseases worldwide are believed to be associated with biofilm formation (Jamal et al., 2018), this is one of the main virulence factors of numerous bacterial species such as *Staphylococcus* sp., *Pseudomonas* sp., *Klebsiella* sp., or *Escherichia coli*. Biofilms are bacterial communities that are protectively surrounded by an extracellular matrix, mainly consisting of polysaccharides and proteins (Flemming and Wingender, 2010). Such biofilms exist ubiquitously in commensal, opportunistic, pathogenic or hypervirulent bacteria (Sydow et al., 2022) and are important for initial or permanent colonization. Within this protective matrix bacteria are largely unaffected by external conditions, including treatments with antibiotics. Substances that can reach and combat planktonic cells are often ineffective against cells within the matrix, especially since the metabolism of these cells is reduced (Mah and O'Toole, 2001). The effect of different compounds on biofilm formation has been analyzed in several studies. For example, (Manner et al., 2013). demonstrated the efficacy of flavonoids against *S. aureus* biofilm formation in a broad screening (Manner et al., 2013). Matilla-Cuenca et al. (2020) focused on flavonoids that target the staphylococcal biofilm-associated protein (BAP) and found quercetin, myricetin and scutellarein to be particularly effective. Pruteanu et al. (2020) also showed that flavonoids can inhibit biofilm formation in *E. coli* and other gram-negatives, particularly by preventing the assembly of the amyloid structures CsgA and CsgB. For epigallocatechin gallate (EGCG), an abundant compound in green tea, Serra et al. (2013) found pronounced biofilm inhibition with respect to *E. coli* macro colonies. In addition to that, phenolic substances are mostly discussed as active components in extracts used for the inhibition of biofilms of various bacterial species (Lee et al., 2013; Bazargani and Rohloff, 2016; Nostro et al., 2016; Sakarikou et al., 2020). However, the underlying mechanisms of action of these different compounds are only known to a limited extend. Yet knowledge about the mechanisms of action is crucial for further drug development, firstly, in order to be able to explain and predict undesirable side effects of the compounds. Secondly, after clarifying the actual target of the compounds, mechanistic knowledge is necessary to enable optimization of the effects by using medicinal chemistry methods to derivatize the substances involved in the effect or to meaningfully expand the spectrum of application without broad screening (Atanasov et al., 2021).

As mentioned above, the mechanism of action for biofilm inhibition is largely unknown for many natural compounds, except for some flavonoidic compounds. Here, the disruption of the formation of the extracellular matrix (mainly curli) by influencing the regulatory gene *csgD* and upstream sigma factors has been reported as essential (Lee et al., 2013; Serra et al., 2016; Hengge, 2020). Furthermore, an increase in bacterial motility is associated with reduced biofilm formation and the genes *cheA, tar, motA* and/or the master regulators *flhD* and *fliA* are particularly noteworthy with respect to the switch from a sessile to a motile lifestyle (Wood et al., 2006; Ito et al., 2009; Hengge, 2020). In addition, the influence of quorum sensing is crucial for biofilm formation (Paluch et al., 2020) and genes like *lsrA, luxS* and *luxR* are particularly relevant (Lee et al., 2013). Finally, metabolic processes (Lindgren et al., 2014; Jakubovics et al., 2015) and transport pathways (Nishino and Yamaguchi, 2001; Herzberg et al., 2006; Ito et al., 2009) are also important for biofilm formation.

**Figure 1:**
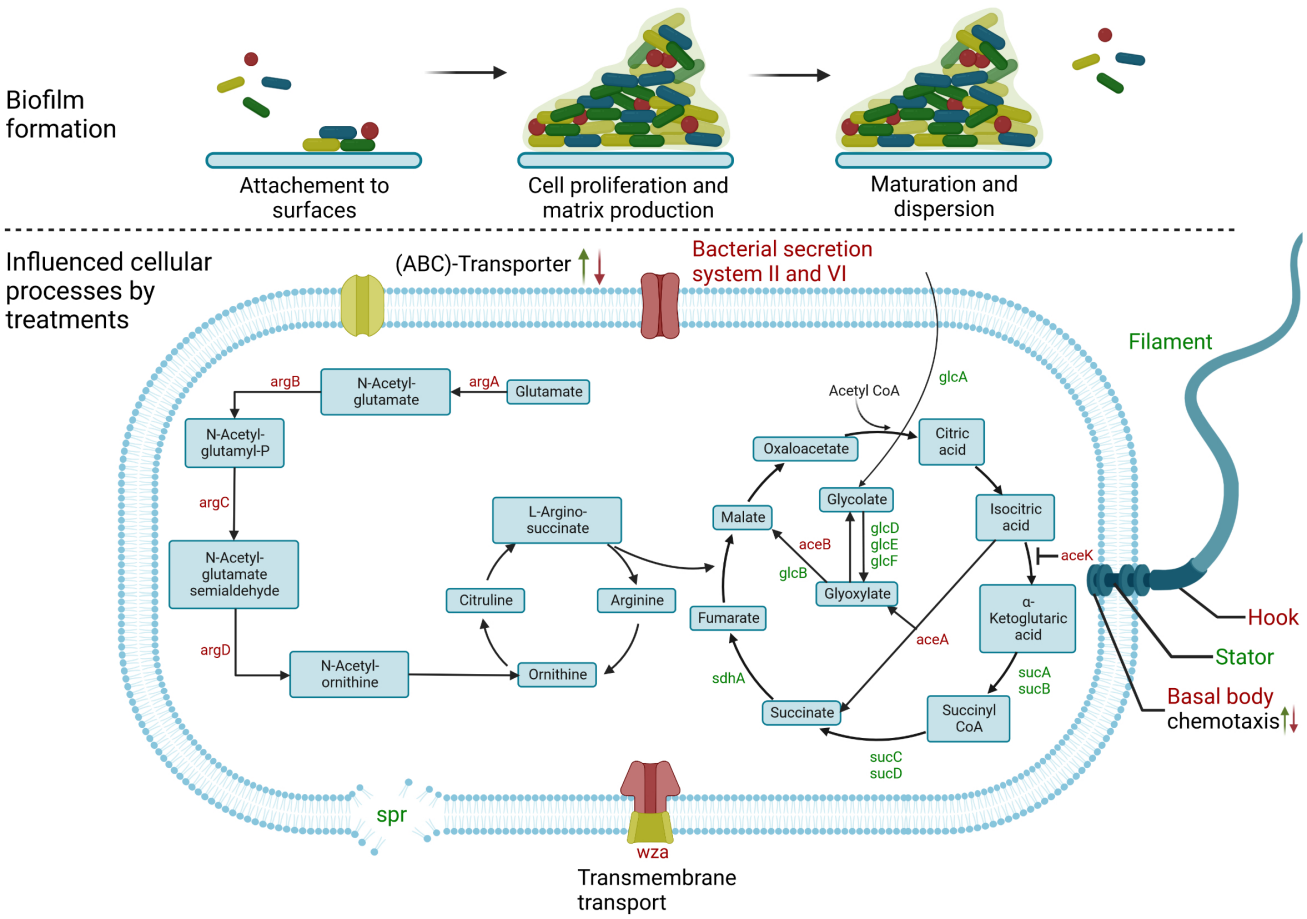
Scheme of biofilm formation in *E. coli.* **(Upper)** General steps of bacterial biofilm formation. **(Lower)** Signaling pathways and genes affected after treatment of *E. coli* biofilm formation in this study. Up arrow indicates up-regulation; down arrow indicates down-regulation. In some cases, both were detectable, depending on the treatment or genes involved. Gene names highlighted in green are also up-regulated, while genes highlighted in red are down-regulated. Created with BioRender.com.

In a recent study we were able to identify natural or natural-like biofilm inhibitors of *E. coli* biofilm formation by employing a machine-learning-based prediction model that used a combined prediction approach and then verifying the results by phenotypic biofilm formation tests (Stepanov et al., 2022). Such computational predictive models reduce both the time and financial costs required for the discovery of new compounds. Moreover, such models can also provide insights into structure–activity relationships (Neves et al., 2018). It is noteworthy that we identified compounds which, despite being chemically very heterogeneous, led to an identical biofilm inhibition phenotype. Based on the reports of several pathways involved in biofilm formation and the outcome of our phenotypic screening, we hypothesize that a complex interplay of pathways is involved in the effective inhibition of biofilm formation (Figure 1).

**Figure 2:**
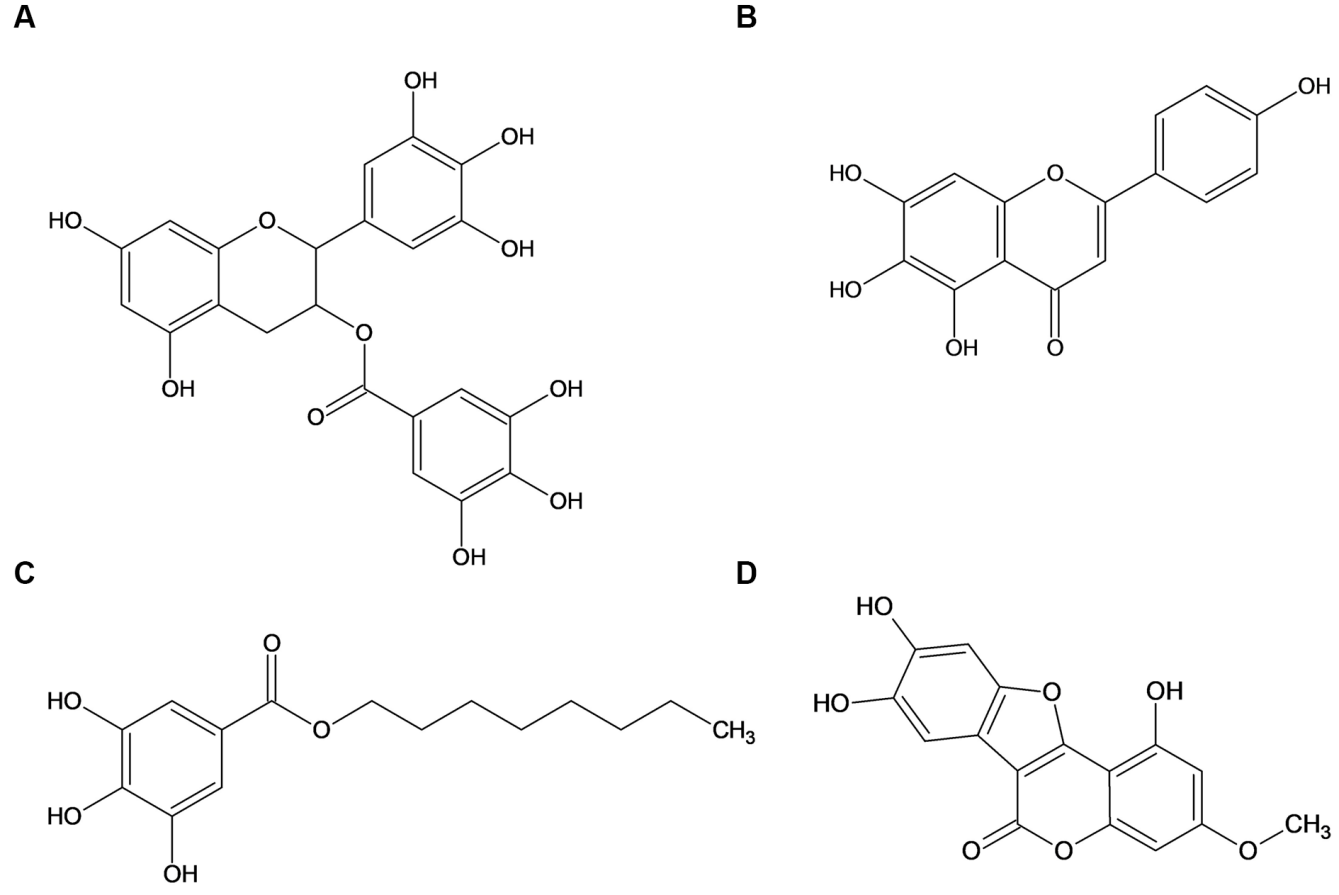
Structure of the investigated natural phenolic compounds epigallocatechin gallate **(A)**, scutellarein **(B)**, octyl gallate **(C),** and wedelolactone **(D)**.

In this study, we thus investigated four different natural phenolic compounds (see Figure 2), which all showed strong biofilm inhibitory activity against *Escherichia coli* PBIO729 (extraintestinal pathogenic *Escherichia coli* [ExPEC], ST131, multidrug-resistant [MDR], Extended-spectrum *β*-lactamases [ESBL]-producing, CTX-M-15) but are structurally different. The international, high-risk clonal *E. coli* lineage sequence type (ST131) has become the major cause of extraintestinal infections by *E. coli* (Liu et al., 2018) and therefore PBIO729 was used as the model. Epigallocatechin gallate served as a reference compound as it has been widely described, scutellarein was identified using classic broad wet-lab screening and octyl gallate as well as wedelolactone were included because they were identified using machine-learning-based prediction models in combination with phenotypic verification (Stepanov et al., 2022). To gain a broad holistic overview of which pathways are involved, we combined phenotypic with RNA sequencing analyses (RNA-Seq) and real-time (RT)-qPCR analyses to analyze the impact of four different compounds on biofilm formation on the transcriptomic level (Yung et al., 2016; Zhao et al., 2018; Seneviratne et al., 2020).

## 2. Materials and methods

### 2.1. Chemicals

Four natural or naturally derived compounds were used for all experiments performed. Epigallocatechin gallate (EGCG) and octyl gallate were purchased by aablocks (San Diego, California, US), wedelolactone and scutellarein from ChemFaces (Wuhan, Hubei, PRC). Stock solutions of 10 mg ml^−1^ were prepared with DMSO and stored at −20°C.

Luria Bertani (LB) medium and Müller-Hinton medium II (MH-II) was prepared according to the manufacturer’s recommendations (Carl Roth GmbH, Karlsruhe, DE).

Dyes used for long term macro colony experiments (Congo red, Coomassie Brilliant Blue G250) were acquired from Carl Roth and Calcofluor White M2R came from Merck KGaA, Darmstadt, DE. Congo red agar consisted of Bacto™ Tryptone (10 mg ml^−1^, Becton, Dickinson and Company, Sparks, US), Bacto™ Yeast Extract (5 mg ml^−1^, Becton, Dickinson and Company) and Span agar (18 mg ml^−1^, H. Carroux, Marschacht, DE) referring to Schaufler et al. (2016). The agar was supplemented with sodium salt (85.5 mM) and, if required, with Congo red (71.77 μM) as well as Coomassie Brilliant Blue G250 (30.27 μM) dissolved in ethanol or Calcofluor White M2R (43.62 μM) dissolved in water.

Chemicals for the RNA-isolation according to the RNAsnap™ protocol of Stead et al. (2012) included Ethylenediaminetetraacetic acid (EDTA, 5.26 mg ml^−1^), Sodium dodecyl sulfate 10% (SDS, 2.5 μl ml^−1^), 2-Mercaptoethanol (2-ME, 0.01 ml ml^−1^) in formamide RNA-grade and were obtained by Carl Roth as well as Guanidinium thiocyanate and isobutanol. Co-Precipitant Pink was from meridian bioscience® (Memphis, Tennessee, US) and RNAse-free water came from Invitrogen Thermo Fisher Scientific (Waltham, Massachusetts, US).

### 2.2. Bacterial strain

A clinical isolate of a multidrug-resistant ESBL-producing *E. coli* strain [PBIO729, originating from the urinary tract infection of a dog, CTX-M-15, resistance genes: blaTEM-1, blaOXA-1, tet(A), tet(R), aadA, aac(6′)-ib-cr] showing strong curli and cellulose production capacities was used in this study (Schaufler et al., 2016; Gerschler et al., 2022). The whole genome sequence of the strain can be obtained from NIH Sequence Read Archive ERR163891. PBIO729 belongs to the international high risk clonal lineage ST131 and our previous studies showed its close phylogenetic relationship to clinical strains from humans and animals as well as commensal strains from wildlife (Schaufler et al., 2019). The bacterium was stored in cryovials containing 20% glycerol, at −80°C. Before use, a fresh bacterial suspension was prepared from an overnight culture on LB-agar-plates in LB-medium. The inoculum of one colony in 5 ml medium was incubated for 12 h at 37°C under shaking conditions with 200 rpm.

### 2.3. Biofilm inhibition assay

**Figure 3:**
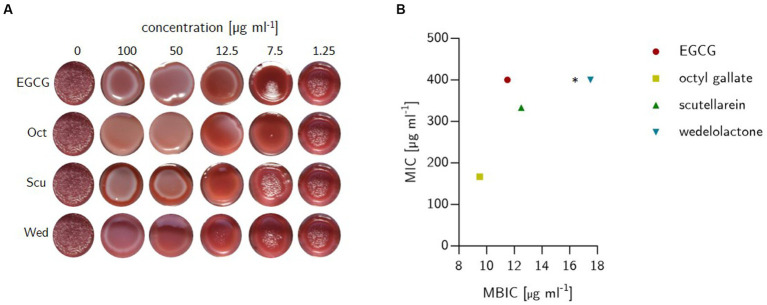
**(A)** Outcome of biofilm inhibition experiments on solid span agar stained with Congo red and Coomassie Brilliant Blue G250 in 24-well plates. The bacteria (*E. coli*) were grown for 48 h at 28°C. Treatments were filled in wells prior to the addition of liquefied agar and comprised epigallocatechin gallate (EGCG), octyl gallate (Oct), scutellarein (Scu) and wedelolactone (Wed) at decreasing concentrations (100, 50, 25, 12.5, 7.5, and 1.25 μg ml^−1^). The plates contained one well without anything added (sterility control) and growth controls with 10 μl (final concentration 1%) DMSO. **(B)** Minimal biofilm inhibitory concentration (MBIC) and minimal inhibitory concentration (MIC) of natural compounds against *E. coli* PBIO729. MIC was investigated in MH-II-broth and detected visually after growth at 37°C for 24 h, while MBIC was determined in solid agar (see left picture). The dots depict the average of three (MIC) or five (MBIC) independent replicates. * The MIC was not detectable within the chosen concentration.

The long-term macro colony growth assay was performed as recently described by Stepanov et al. (2022) to detect biofilm inhibition by screening for the production of curli fibers and cellulose as these are essential components of *E. coli* biofilm formation (Stepanov et al., 2022). One milliliter Congo red agar was filled in the wells of 24-well plate containing either test substances (100, 75, 50, 25, 12.5, 10, 7.5, 5, 2.5, 1.25 μg ml^−1^) or solvent control (SvC DMSO [<2%]). One well without any content was used as a growth control (GC) accounting for 100% of the biofilm production. Five microliter of bacteria suspension with an optical density of 0.5 (approx. 8∙10^7^ CFU ml^−1^) were dropped into the center of each well. The prepared plates were incubated under aerobic static conditions for 48 h at 28°C. Inhibition of biofilm formation was determined visually based on the colony phenotypes. There was deemed to be no inhibition of biofilm formation if the colonies had the same structure as the growth control (red and rough) after treatment. If the colonies looked smooth and only slightly red, biofilm inhibition was present (see Figure 3A). The experiment was conducted with five independent biological replicates, and the minimal biofilm inhibitory concentration (MBIC) was calculated as the mean of five followed by a statistical one-way ANOVA test with Dunnett’s multiple comparison using GraphPad Prism 10.0.1 software.

The same assay conditions were used for the quantification of the biofilm inhibition, but instead of Congo Red and Coomassie Brilliant Blue, Calcofluor White M2R was added to stain the cellulose. Subsequent to incubation, fluorescence intensity was measured using the Omega Fluostar spectrophotometer (BMG LABTECH GmbH, Ortenberg, DE). The excitation wavelength was set at 400 nm and emission was measured at 480 nm with a fixed Gain of 500. A well scanning method with 15 × 15 points was used, and a manually selected matrix of 6 × 6 values formed the basis for an average detection of biofilm formation in the colonies. This is necessary because the colonies do not always have the same position in the well and to ensure, that only the colony is detected without any agar. The test was performed three times independently and the intensity was calculated as mean ± standard deviation. For statistical evaluation a two-way ANOVA test with Dunnett’s multiple comparison was performed with the control DMSO using GraphPad Prism.

### 2.4. Image analysis

Image analysis was performed to objectify the results of the biofilm inhibition assays on Congo red agar. The R programming language (v. 4.3.1) was used to analyze the image data using the RKWard (v.0.7.5z + 0.7.6 + devel1) programming environment (Rödiger et al., 2012). The imager package (Schneider et al., 2019) was used to obtain the data matrices from the images. R scripts were created for all steps. For details in processing see Supplementary Data S3.

### 2.5. Minimum inhibitory concentration

Determination of MIC of the investigated natural compounds was performed with a serial broth dilution method, as described previously (Buchmann et al., 2022). According to Clinical and Laboratory Standards Institute (CLSI) guidelines (CLSI, 2012) EGCG, octyl gallate, scutellarein and wedelolactone were tested using MH-II-medium, which had been prepared according to the manufacturers’ recommendations, in clear 96-well plates (sterile F, Carl Roth). Experimental concentrations ranged from 400 to 6.125 μg ml^−1^ obtained by serial two-fold dilution of 8 μl stock solution of each compound in 180 μl medium (row A to row G). Ten microliter of bacterial suspension with an optical density of 0.1 were added to each well, except for row H, which served as a sterility control. A growth and solvent control with DMSO (final concentration was never more than 4% and did not affect bacterial growth) were performed in parallel. After incubation for 24 h at 37°C, the MIC value was recorded visually as the minimum compound concentration at which no visible growth occurred. The determination of the MIC was carried out with three independent biological replicates and was calculated as the mean of three.

### 2.6. Scanning electron microscopy

A hydrophilic polycarbonate filter (0.2 μm pore size, Merck Millipore) was placed on span agar containing the control (1% DMSO) or compounds (100 μg ml^−1^). As described above, 5 μl of a bacteria suspension was dropped onto the filter and incubated for 48 h at 28°C. The filter with the grown colony was triangular segmented and a segment with the edge of the colony was transferred into the fixative (1% glutaraldehyde, 4% paraformaldehyde, 0.2% picric acid in 20 mM HEPES buffer). Fixation was carried out for 1 h at room temperature (RT) and samples were then stored at 4°C until further processing. Samples were washed with washing buffer (100 mM cacodylate buffer, 1 mM CaCl_2_; pH 7) three times for 10 min each time, treated with 2% tannic acid in washing buffer for 1 h at RT, and washed again with washing buffer three times for 15 min each time. Then, the samples were dehydrated in a graded series of aqueous ethanol solutions (10, 30, 50, 70, 90, 100%) on ice for 15 min each step. Before the final change of 100% ethanol, the samples were allowed to reach RT and then critical point-dried with liquid CO_2_. Finally, samples were mounted on aluminum stubs, sputtered with gold/palladium and examined with a field emission scanning electron microscope Supra 40VP (Carl Zeiss Microscopy Deutschland GmbH, Oberkochen, Germany) using the Everhart-Thornley SE detector and the in-lens detector in a 70:30 ratio at an acceleration voltage of 5 kV. All micrographs were edited by using Adobe Photoshop CS6.

### 2.7. RNA isolation

The RNASnap method (Stead et al., 2012) with minor modifications was used to extract RNA from biofilm colonies of PBIO729 with and without treatment. Macro colonies were completely scraped off the agar of the well with the aid of an inoculation loop and transferred into a 1.5 ml reaction tube containing acid-washed glass beads (≤106 μm, Merck KGaA, Darmstadt, DE). The colony was suspended in 500 μl formamide solution and shaken intensively for 30 s using a test-tube shaker (lab dancer, VWR International GmbH, Darmstadt, DE) in order to remove externally adherent RNAses on the biofilm colonies. The tubes were then centrifuged at 16000 × *g* for 3 min at room temperature and the supernatant was discarded. The pellet was resuspended together with the glass beads in 500 μl RNA extraction solution and then treated in the bead beating grinder and lysis system FastPrep™-24 benchtop homogenizer (MP Biomedicals Germany GmbH, Eschwege, DE) at 5 m s^−1^ three times for 30 s each. The subsequent cell lysis was carried out in a heating block at 95°C for exactly 7 min. Afterwards, centrifugation was repeated for 5 min at 16000 × *g* without cooling. The supernatant was carefully transferred into a new 2 ml reaction tube without damaging the gelatinous pellet. Fifty microliter of 3 M sodium acetate solution was added to the 500 μl of supernatant and mixed intensively. Then 5 μl of Co-Precipitant Pink, 500 μl of 5 M guanidinium thiocyanate solution and 0.9 ml of isobutanol were added. After mixing well, the solution was incubated at room temperature for 10 min, then centrifuged at 13000 × *g* for 5 min. The supernatant was discarded and the remaining faintly pink colored pellet was washed three times with 2 ml of 75% ethanol. Finally, all liquid was removed and the pellet was collected in 30 μl RNAse-free water. Following this step, the quality (260/280 ratio, and 260/230 ratio) and concentration of the RNA samples were determined using the Omega Fluostar spectrophotometer. Additionally, the integrity of RNA was checked with the 2,100 Bioanalyzer (Agilent Technologies, Inc., Santa Clara, CA, US).

### 2.8. RNA sequencing

RNA was isolated from two biological replicates as mentioned above and shipped to LGC Genomics GmbH (Berlin, DE). Sequencing was performed using an Illumina NextSeq 550/550 v2 machine, producing 75 bp single-end reads. The obtained RAW data of all libraries for each sequencing lane were demultiplexed using the Illumina bcl2fastq v2.20 software (Illumina, 2019). FastQC v0.11.9 (Andrews, 2019) reports containing read quality metrics were created and stored along with the FASTQ files (see Supplementary Data S2).

### 2.9. Data analysis of RNA-Seq experiments

mRNA sequencing data received from LGC as RAW data were processed with different bioinformatics tools. First, the adapters were each clipped using trimmomatic v.0.39 with Illuminaclip TruSeq3-SE:2:30:10, Leading and Trailing 3, Slidingwindow:4:15 and a minimum length of 36 base pairs. A FASTQC report was then generated to determine the quality of sequenced and trimmed reads. Subsequently, the rRNA (16, 23, 5 s) was filtered and removed using Ribopicker v.0.4.3 with at least 80% coverage and 90% identity (Schmieder et al., 2012). After that, the alignment of the reads with the whole genome sequence of PBIO729 (ERR163891) was examined with Bowtie2 v.2.3.5.1 (Langmead and Salzberg, 2012) as well as BWA v.0.7.17 (bwa mem) (Li, 2013). Successfully mapped reads were converted from SAM to BAM with samtools and sorted with the same program (Li et al., 2009) before being counted with htseq-count v.0.13.5 (Anders et al., 2015) and processed in the final step with R/Bioconductor packages DESeq (Anders and Huber, 2010) and edgeR (Robinson et al., 2010), resulting in the differentially expressed genes presented as log_2_-fold changes (Log_2_FC). For the most part, genes with LFC ±1.5 and adjusted value of *p*s ≤0.01 were used for further analyses, in accordance with the literature (Peart et al., 2005; Patterson et al., 2006; Raouf et al., 2008); it is stated when this was not the case. The calculation of DEGs resulted in similar counts with both mappers and tools (data not shown).

### 2.10. Gene expression analysis and functional characterization

To determine the functionality of the differentially expressed genes, the transcript sequences were analyzed with blastp provided by NCBI. The most probable hit was taken in each case, if the coverage and identity rate were both 100%. The blastp analyses were done for *E. coli* K-12 as reference strain as well as for the ST131 strain *E. coli* EC958 (NCBI:txid941322). Gene ontology enrichment analysis was performed using BioCyc pathway tools (Keseler et al., 2021). The calculation is based on the statistical method Fisher exact with Benjamini–Hochberg correction. If suitable, the first 30 GO terms with a value of *p* less than 0.1 were considered, otherwise all GO terms were taken. For the KEGG pathway analysis the KEGG Mapper (Kanehisa and Sato, 2020) was used. Voronoi diagrams were created using Proteomaps by Bionic Visualizations (Otto et al., 2010; Liebermeister et al., 2014).

### 2.11. RT-qPCR

For reverse transcriptase real-time PCR, samples were gained as per the description for RNA-isolation. The RNA was diluted with RNAse-free water and 50 ng was used with HotScriptase RT Master mix (Genaxxon bioscience GmbH, Ulm, DE) according to the manufacturer’s instructions. The 96-well plates for qPCR (Genaxxon) contained 12.5 μl master mix, 1.25 μl primer (forward and reverse 10 μM each) and 10 μl sample. The primers are listed in Supplementary Table S1. The temperature regime in Lightcycler96 (Roche AG, Basel, CH) started with a preincubation at 80°C for 60 s, followed by 420 s at 60°C, before ending at 95°C for 180 s. The actual PCR comprised a three-step amplification with 45 cycles of 95°C for 10 s, 75°C for 15 s and 70°C for 60 s. The regime terminated with high resolution melting starting at 95°C for 60 s, then 40°C for 60 s, and subsequently 65°C for 1 s and continuous heating to 97°C with 0.07°C s^−1^. The data were analyzed using LightCycler®96 Software with a detection threshold of 0.2. Target gene expression was calculated to be relatively normalized to the reference genes *rpoA, rrsa* and *dnaE* with the 2^-(ΔΔct)^-method according to Livak and Schmittgen (2001).

The experiment was repeated three times (three independent biological replicates) with two technical replicates for each gene and the average of fold gene expression was calculated. The results were statistically analyzed with two-way ANOVA and Dunnett’s multiple comparison test using GraphPad Prism.

### 2.12. Motility assay

Following the procedure of Barker et al. (2004) the swimming behavior on semisolid LB agar plates (LB medium containing 0.25% agar) was investigated. Macro colonies (treated and untreated) grown on span agar in 24-well plates were scraped off with a sterile toothpick and transferred into the center of petri dishes filled with 20 ml of freshly prepared (60°C) motility agar, which was allowed to cool for 30 min. After 45 min drying time the plates were incubated at 37°C for approx. 18 h. Then the diameter of colonies was measured. Three independent biological replicates were performed and the average diameter of swimming and the standard deviations were calculated.

## 3. Results

### 3.1. Biofilm inhibition and minimal inhibitory concentration

**Figure 4:**
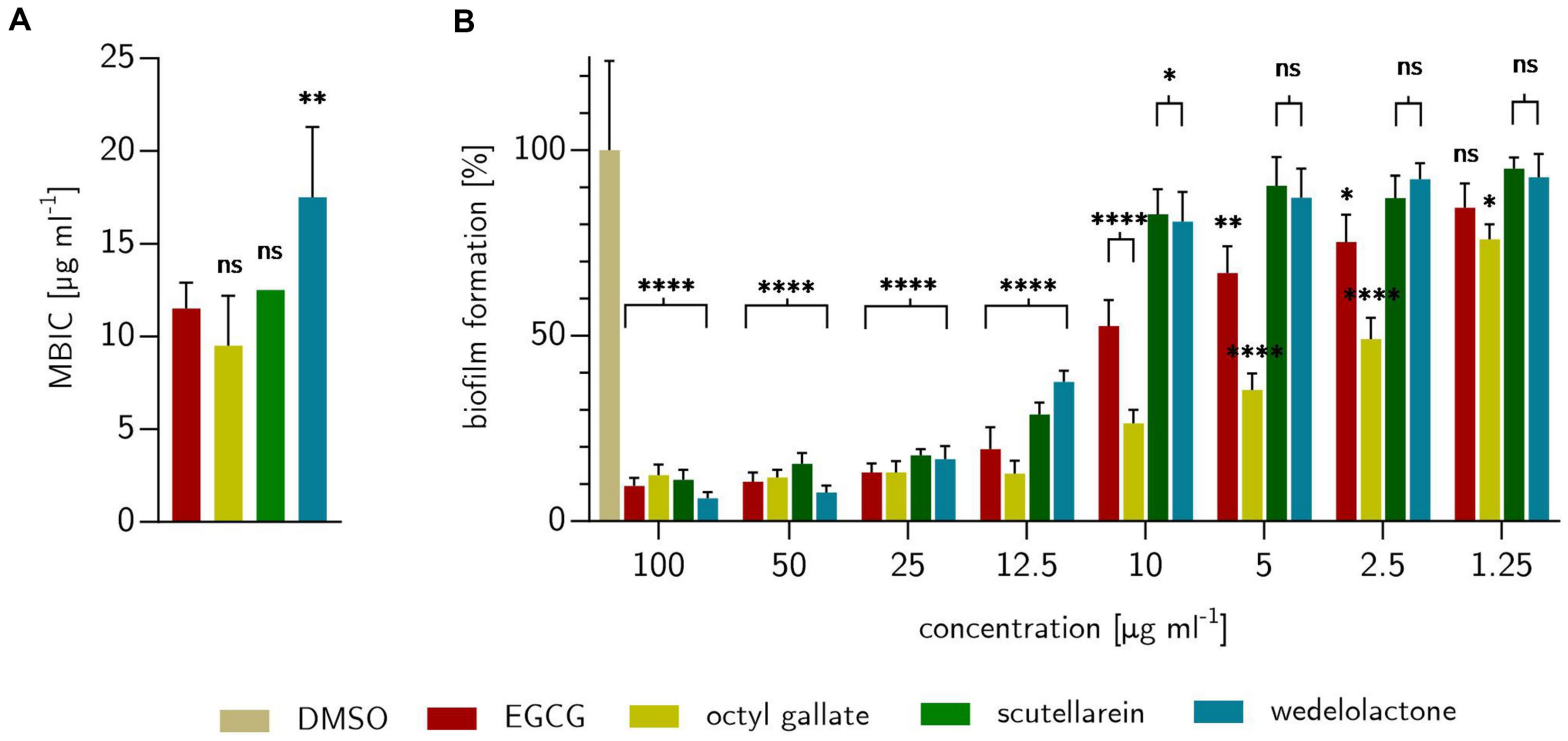
Effects of treatments with EGCG, octyl gallate, scutellarein or wedelolactone on *E. coli* PBIO729. **(A)** Treatment concentration for inhibition of visual detectable biofilms after 48 h on stained Congo red Coomassie-brilliant blue agar compared to positive substance EGCG. **(B)** Percentage of formed biofilms, expressed as cellulose, detected fluorometrically with Calcofluor White M2R on solid agar after 48 h. The comparison shown is for DMSO as 100% biofilm formation. Statistical analysis was performed with GraphPad prism 10.0.1 as one-way ANOVA **(A)** or two-way ANOVA **(B)** always with Dunnett’s multiple comparison. Experiments were repeated three times. Stars indicate value of *p*s: *p ≤ 0.05, **p ≤ 0.01, ***p ≤ 0.001, ****p ≤ 0.0001, *ns* > 0.05.

To demonstrate the biofilm inhibitory properties of the natural compounds EGCG, octyl gallate, scutellarein and wedelolactone in comparison to the solvent control DMSO, a macro colony assay was performed on Congo red agar in a 24-well format. The investigated substances showed an intensive biofilm inhibition at 100 μg ml^−1^ (see Figure 3A). Compared to the growth and solvent control, macro colonies were no longer structured (reduction of cellulose formation), but completely smooth and largely decolorized (which indicates a reduction in the binding of amyloids to Congo red). Octyl gallate even caused complete decolorization. The complex structure of the biofilms was altered in particular the cellulose-related structures during treatment and, to a large extent, the amyloids like curli were reduced. The visually detected minimal biofilm inhibitory concentration (MBIC) was 11.5 ± 1.4 μg ml^−1^ for EGCG, 12.5 μg ml^−1^ for scutellarein and 17.5 ± 6.8 μg ml^−1^ for wedelolactone. For octyl gallate the MBIC was 9.5 ± 2.7 μg ml^−1^ (see Figures 3B, 4A). This means, that the inhibition activity of wedelolactone was significantly lower than that of the control compound EGCG. These results were also confirmed by the quantification of cellulose as component of the extracellular matrix (ECM). The degree of biofilm inhibition could also be determined with this fluorescence-based assay. As shown in Figure 4B, all treatments inhibited the biofilm formation almost completely at concentrations above 25 μg ml^−1^. Octyl gallate acts even stronger than EGCG over all investigated concentrations and the others have smaller effects but are significant at concentrations above 12.5 μg ml^−1^ compared to the untreated colonies.

We did not observe a general growth inhibition as the colonies showed comparable expansions after 48 h (see Figure 3A). These observations were confirmed by MIC examinations that showed no antibacterial effects for the compounds at concentrations of interest for biofilm inhibition (up to 100 μg ml^−1^). Overall, the only detectable inhibition of bacterial growth was for octyl gallate (167 ± 58 μg ml^−1^) and scutellarein (333 ± 116 μg ml^−1^) (see Figure 3B).

### 3.2. Image analysis

Bioimage informatics can be used to obtain quantitative information from image data (Schneider et al., 2019). The biofilm inhibition experiments strongly suggested that the treatments differed in their surface area also in a concentration dependency (see Figure 4B). The image analysis based on the parameters of intensity and surface conditions confirmed these observations. For more details see Supplementary Data S3.

### 3.3. Scanning electron microscopy

**Figure 5:**
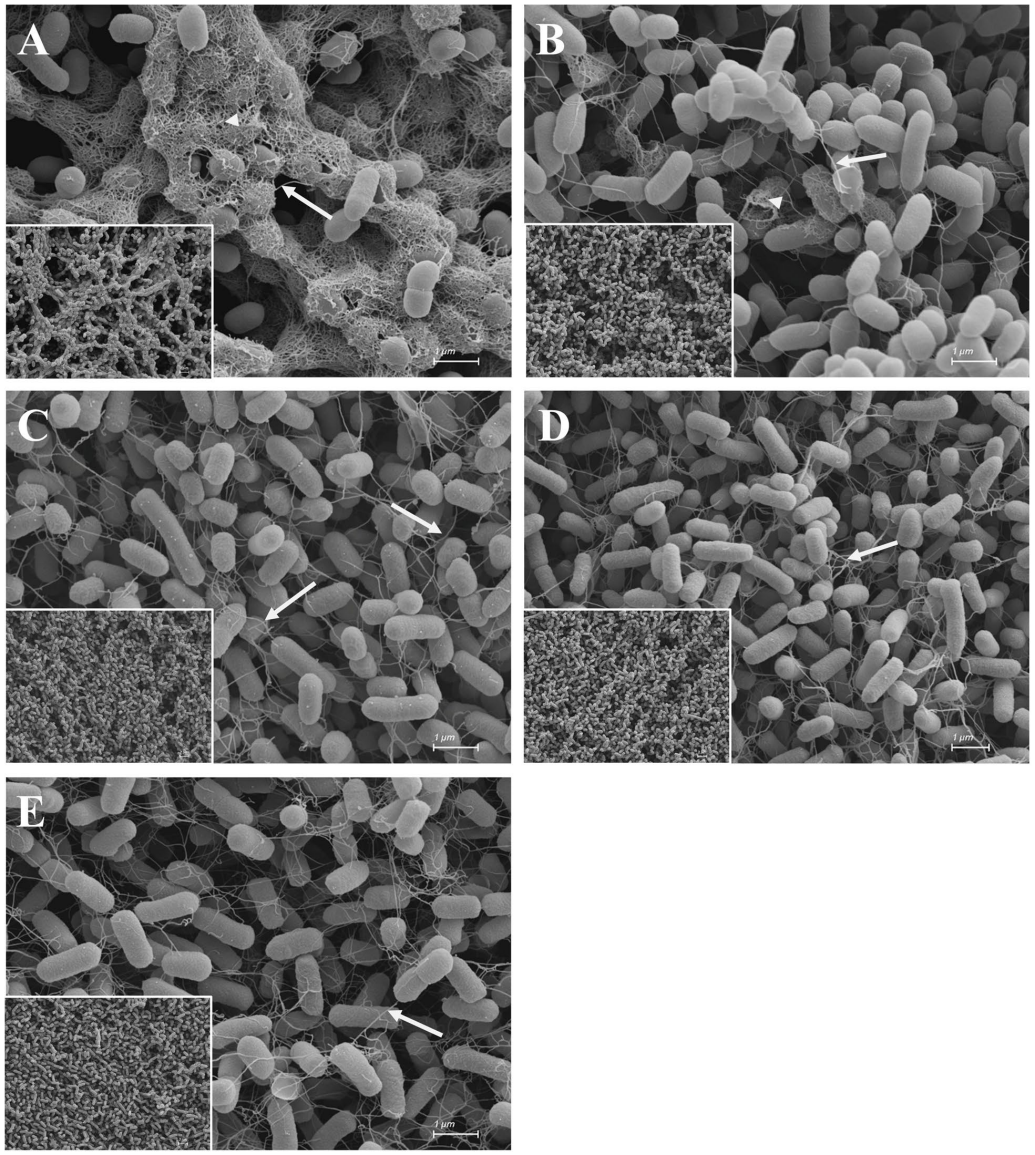
Scanning electron micrographs of DMSO **(A)**, EGCG **(B)**, octyl gallate **(C)**, scutellarein **(D),** and wedelolactone **(E)** treated macro colonies of *E. coli* PBIO729. Macro colonies were grown on a 0.2 μm polycarbonate filter on span agar for 48 h at 28°C. Inset micrographs (left corner) are lower magnifications (at 2500x) giving an overview of the surface structure (for full size see Supplementary Figure S1). Arrows show flagella. Arrowheads show a dense structure around cell bodies originating from curli/fimbriae production. Scale bars = 1 μm. Scale bars of the inset micrographs = 2 μm.

Differences in biofilm formation were also visualized by scanning electron microscopy. The biofilm of cells grown under control conditions exhibits a dense matrix consisting of fibers like curli and/or further fimbriae (Pereira et al., 2010; Lee et al., 2011; Serra et al., 2013; Kleta et al., 2014) which encase the cells (Figure 5A). The bacterial cells themselves are identical in shape and size to those in the untreated control. In part, large gaps between the cells are present (inset micrograph; Figure 5 and Supplementary Figure S1). While curli/fimbriae covered cells are still visible in the EGCG treated biofilm (Figure 5B), albeit greatly reduced, these short fibers are almost no longer present in the biofilms treated with octyl gallate, scutellarein or wedelolactone (Figures 5C–E). Under these conditions cells are mainly connected by flagella. Compared to the control, the general architecture of the biofilm treated with octyl gallate, scutellarein or wedelolactone appears smoother as the gaps are more or less absent.

### 3.4. RNA-isolation and –sequencing

As the treatment of the bacteria with structurally different compounds led to biofilm inhibition with an almost identical phenotypic appearance, RNA-Seq was performed to identify the pathways that are involved under treatment with the investigated compounds. The RNA yields after extraction can be found in Supplementary Table S2.

About 16 million raw reads and 14 million cleaned reads were generated for the DMSO control. For the treatments, the read count of the raw data was 15–17 million and for the cleaned data 13–15 million (Supplementary Table S2). Both the unprocessed and the processed reads met the recommended quality requirements for RNA-Seq analyses (Sheng et al., 2017). The principal component analysis of the sequencing data obviously showed that the treatments were significantly different from the control group, and that the biological replicates of the treatments clustered, therefore, comparable results could be assumed (Supplementary Figure S2).

**Figure 6:**
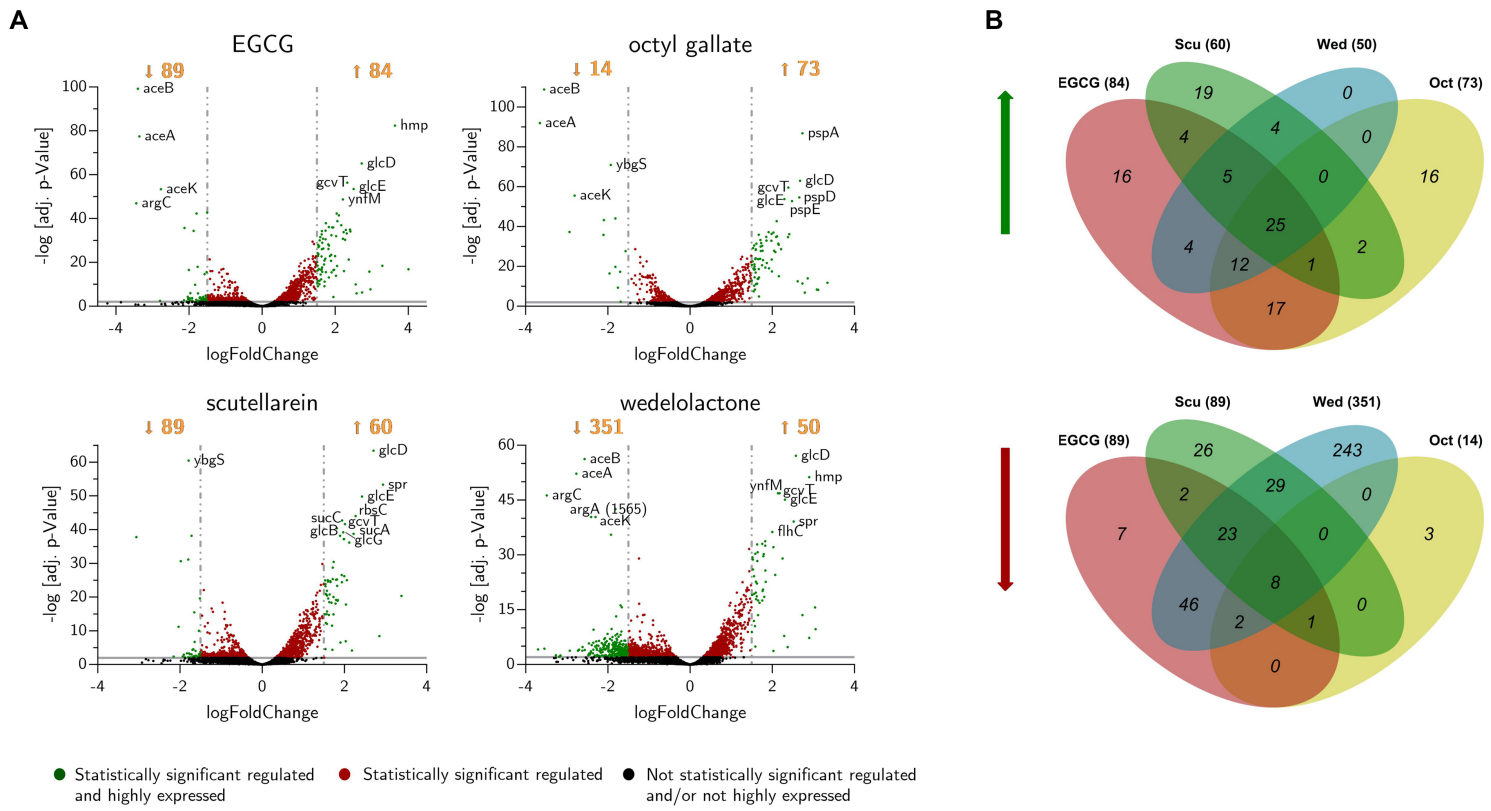
**(A)** Volcano-plot of differentially expressed genes in biofilms of *E. coli* PBIO729 after treatment with epigallocatechin gallate (EGCG), octyl gallate, scutellarein or wedelolactone and incubation for 48 h at 28°C. Statistically significant genes, plotted above the gray line (adj. value of *p* ≤ 0.01), are displayed in red or green. The green ones represent statistically significant regulated and highly expressed genes. The most significantly affected genes are labeled with their name. **(B)** Venn diagram of the numbers of up- (left) and down-regulated (right) genes after treatment with biofilm inhibitors. The diagram shows genes that are regulated with at least a Log_2_-fold change of ±1.5 and an adjusted value of *p* of 0.01. Sums of regulated genes are shown in brackets behind treatment indicators. EGCG, epigallocatechin gallate; Scu, scutellarein; Wed, wedelolactone; Oct, octyl gallate.

Using DESeq2, a total of 515 genes were found to be differentially expressed across all treatments, including 390 down-regulated and 125 up-regulated unique genes. Most of the regulated genes were compound-specific and only 33 identical genes were affected by all four compound treatments (Figure 6B and Table 1). Based on the parameters mentioned in the methods, genes were identified that were statistically significantly differentially expressed compared to the control group (Table 1 and Figure 6A). For all conditions, we found up-regulation of genes involved in bacterial flagellar synthesis, chemotaxis, and the tricarboxylic acid cycle, as well as extensive up-regulation in glyoxylate and dicarboxylate metabolism. In addition, we detected an almost complete down-regulation of all genes involved in arginine biosynthesis.

**Table 1:** Differentially expressed genes after treatment of *E. coli* PBIO729 biofilms on solid stained agar with EGCG, octyl gallate, scutellarein and wedelolactone after 48 h.

Gene ID	Gene name	Gene function
DOPLEBMP_00539	*flhC*	Flagellar transcriptional regulator FlhC
DOPLEBMP_00540	*flhD*	Flagellar transcriptional regulator FlhD
DOPLEBMP_00626	*fliC*	Flagellin, the subunit protein which polymerizes to form the filaments of bacterial flagella.
DOPLEBMP_00670	*hiuH*	5-hydroxyisourate hydrolase
DOPLEBMP_00962	*spr*	Murein DD-endopeptidase MepS/Murein LD-carboxypeptidase
DOPLEBMP_01235	*yffB*	Protein YffB
DOPLEBMP_01633	*gcvH*	Glycine cleavage system H protein, shuttles the methylamine group of glycine from the P protein to the T protein.
DOPLEBMP_01634	*gcvT*	Aminomethyltransferase in the glycine cleavage system
DOPLEBMP_01694	*ansB*	l-asparaginase 2, a high-affinity secreted enzyme
DOPLEBMP_01804	*glcA*	Glycolate permease GlcA for uptake of glycolate across the membrane
DOPLEBMP_01805	*glcB*	Malate synthase G. Catalyzes the condensation and subsequent hydrolysis of acetyl-coenzyme A (acetyl-CoA) and glyoxylate to form malate and CoA.
DOPLEBMP_01806	*glcG*	Protein GlcG
DOPLEBMP_01807	*glcF*	Glycolate oxidase iron–sulfur subunit, component of a complex that catalyzes the oxidation of glycolate to glyoxylate.
DOPLEBMP_01808	*glcE*	Glycolate oxidase subunit GlcE, component of a complex that catalyzes the oxidation of glycolate to glyoxylate.
DOPLEBMP_01809	*glcD*	Glycolate oxidase subunit GlcD, component of a complex that catalyzes the oxidation of glycolate to glyoxylate.
DOPLEBMP_02927	*malE*	Maltose/maltodextrin-binding periplasmic protein, part of the ABC transporter complex MalEFGK involved in maltose/maltodextrin import.
DOPLEBMP_02973	*cidA*	Holin-like protein
DOPLEBMP_02974	*yohK*	Inner membrane protein YohK
DOPLEBMP_03731	*prpB*	2-methylisocitrate lyase, involved in the catabolism of short chain fatty acids via the 2-methylcitrate cycle I (propionate degradation route).
DOPLEBMP_03732	*prpC*	2-methylcitrate synthase, involved in the catabolism of short chain fatty acids via the tricarboxylic acid (acetyl degradation route) and via the 2-methylcitrate cycle I (propionate degradation route).
DOPLEBMP_03734	*prpE*	Propionate-CoA ligase, catalyzes the synthesis of propionyl-CoA from propionate and CoA
DOPLEBMP_04086	*sdhA*	Succinate dehydrogenase flavoprotein subunit. Two distinct, membrane-bound, FAD-containing enzymes are responsible for the catalysis of fumarate and succinate interconversion; the fumarate reductase is used in anaerobic growth, and the succinate dehydrogenase is used in aerobic growth.
DOPLEBMP_04087	*sdhB*	Succinate dehydrogenase iron–sulfur subunit. Two distinct, membrane-bound, FAD-containing enzymes are responsible for the catalysis of fumarate and succinate interconversion; the fumarate reductase is used in anaerobic growth, and the succinate dehydrogenase is used in aerobic growth.
DOPLEBMP_04088	*sucA*	E1 component of the 2-oxoglutarate dehydrogenase (OGDH) complex which catalyzes the decarboxylation of 2-oxoglutarate, the first step in the conversion of 2-oxoglutarate to succinyl-CoA and CO2.
DOPLEBMP_04089	*sucB*	Dihydrolipoyllysine-residue succinyltransferase. E2 component of the 2-oxoglutarate dehydrogenase (OGDH) complex which catalyzes the second step in the conversion of 2-oxoglutarate to succinyl-CoA and CO2.
DOPLEBMP_00257	*ydgI*	Putative arginine/ornithine antiporter. Catalyzes electroneutral exchange between arginine and ornithine to allow high-efficiency energy conversion in the arginine deiminase pathway.
DOPLEBMP_01565	*argA*	Amino-acid acetyltransferase
DOPLEBMP_02843	*argC*	*N*-acetyl-gamma-glutamyl-phosphate reductase. Catalyzes the NADPH-dependent reduction of *N*-acetyl-5-glutamyl phosphate to yield *N*-acetyl-l-glutamate 5-semialdehyde.
DOPLEBMP_02844	*argB*	Acetylglutamate kinase. Catalyzes the ATP-dependent phosphorylation of *N*-acetyl-l-glutamate.
DOPLEBMP_02899	*aceB*	Malate synthase A
DOPLEBMP_03737	*lacA*	Galactoside *O*-acetyltransferase. Catalyzes the CoA-dependent transfer of an acetyl group to the 6-*O*-methyl position of a range of galactosides, glucosides, and lactosides.
DOPLEBMP_03738	*argA*	Amino-acid acetyltransferase
DOPLEBMP_04223	*artJ*	Part of the ABC transporter complex ArtPIQMJ involved in arginine transport. Binds l-arginine with high affinity.

These genes are up-regulated (upper part of the table) or down-regulated (lower part) by all treatments with at least a Log_2_-Fold change of 1.5 and adjusted *p*-value of 0.01. Named gene function is based on KEGG.

### 3.5. RNA analysis

**Figure 7:**
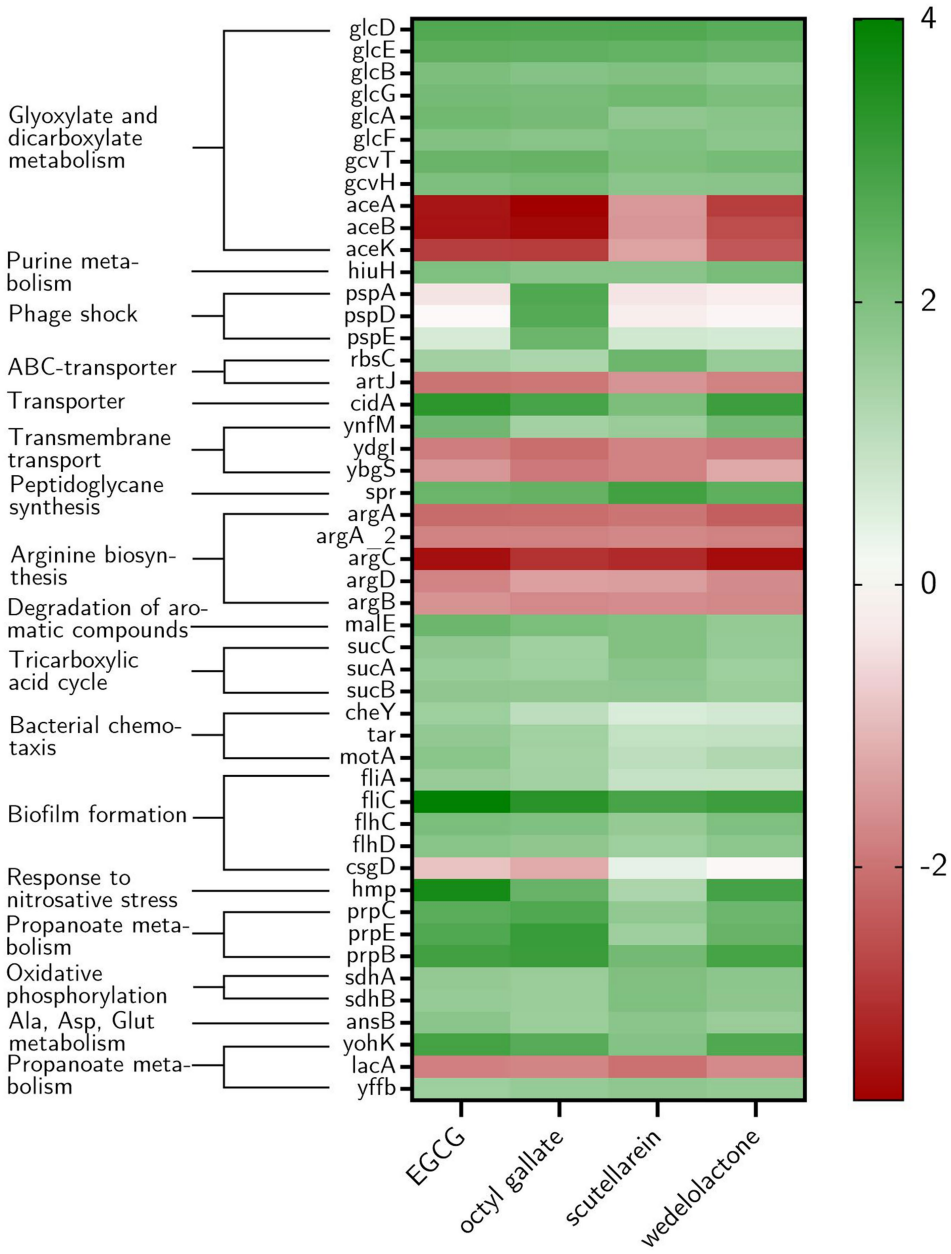
Heat map showing the ten most highly expressed genes in *E. coli* biofilms under different treatment conditions as well as the genes that were regulated by all treatments. Furthermore, genes associated with biofilm formation are displayed if at least one treatment led to differential expression and *csgD* due to the described influence of EGCG on its expression. The log_2_Fold change (compared with the non-treatment DMSO-group) is shown from −4 to 4 (red to green). In addition, the corresponding KEGG pathways that the genes belong to are named.

The amount of differentially expressed genes in PBIO729 after treatment and their annotation to the database including strain EC958 (BioCyc using NCBI:txid941322) is shown in Table 2. To gain more insights into qualitative and quantitative differences in gene expression between the treatments a heat map was created (Figure 7), which displays all 33 genes that were affected under treatment with all four compounds compared to the solvent control, plus the 10 most highly regulated genes for each compound as well as typical biofilm/motility-associated genes of interest, such as *csgD*. Overall, a comparable pattern of regulated genes was present in all four treatments. For example, genes of the glyoxylate cycle including *glcB, glcD, glcE,* and *glcG* were significantly up-regulated across all treatments with Log_2_-fold changes between 1.8 (*glcB* by wedelolactone) and 2.7 (*glcD* by EGCG, octyl gallate and scutellarein) compared to the control. Also noteworthy is the overexpression, for all treatments, of the motility-related genes *fliA, flhC, flhD* and especially *fliC*. The latter was most intensively up-regulated by EGCG (Log_2_FC = 4.0) and least strongly by scutellarein (Log_2_FC = 2.9). The same pattern was observed for the other genes (*fliA*: Scu ≤ Wed ≤ Oct ≤ EGCG, for *flhC*: Scu ≤ Oct ≤ Wed ≤ EGCG, for *flhD*: Scu ≤ Oct ≤ Wed ≤ EGCG and for *fliC*: Scu ≤ Wed ≤ Oct ≤ EGCG). The expression of the genes *cheY, motA* and *tar*, which are involved in bacterial chemotaxis, was also positively influenced by all treatments. Nevertheless, for some compounds, specific pathways that are not present under all treatment conditions seem to be involved, one example being the up-regulation of phage shock protein under the treatment of octyl gallate. Also, differences in the quantity of gene expression were detected for genes like *aceA*, *aceB,* or *aceK*, which were less down-regulated under treatment with scutellarein (Log_2_FC = −1.5, −1.5, −1.3) than, e.g., under treatment with octyl gallate (Log_2_FC = −3.7, −3.5, −2.8). These genes belong to the glyoxylate cycle and encode proteins responsible for glyoxylate synthesis (*aceA*), subsequent malate synthesis *(aceB)* and the connection between TCA cycle and glyoxylate cycle (*aceK*). As mentioned above, genes involved in arginine biosynthesis were down-regulated for all compounds, including *argC* (Log_2_FC = −2.9 to −3.5), and *argA* or *argD*, with average Log_2_-fold changes of −2.0 to −2.3 and − 1.4 to −1.8, respectively. Interestingly, the global regulator *csgD* associated with biofilm formation was neither highly expressed nor significantly altered by any substance (Log_2_FC_EGCG_ = −0.9, Log_2_FC_Oct_ = −1.2; Log_2_FC_Scu_ = 0.4, Log_2_FC_Wed_ = −0.1; *p* ≥ 0.01).

**Table 2:** Differential expressed genes (DEGs) in PBIO729 biofilms after treatment with epigallocatechin gallate (EGCG), octyl gallate (Oct), scutellarein (Scu) or wedelolactone (Wed) and the amount of annotated genes against the genome of *E. coli* EC958 (ST131 lineage) received with blastp by NCBI.

Compound	DEGs	Annotated genes	Annotation rate [%]
		*E. coli* EC958	
EGCG	173	155	90
Oct	87	79	91
Scu	149	119	80
Wed	401	351	88

### 3.6. Pathway analyses

Gene Ontology (GO) analysis allows the classification of the functionality of genes of an organism. It mainly describes existing biological knowledge in terms of three aspects: molecular function, cellular components and biological processes. A gene can be assigned to one or more classes (Ashburner et al., 2000). Supplementary Figure S3 summarizes the results of the GO enrichment for *E. coli* EC958 (ST131). A total of 20 different enriched processes, 18 components and 15 functions were identified. As expected, some of these were affected equally by some treatments, others only by single compounds. For example, all agents interfered with the “tricarboxylic acid cycle,” while octyl gallate and scutellarein in particular altered genes according to the GO term “protein-containing complex,” and scutellarein as well as wedelolactone regulated many genes of particular importance for transporter activities. It is remarkable that GO analysis only showed significant effects concerning terms of components and processes related to bacterial motility for octyl gallate and scutellarein.

**Figure 8:**
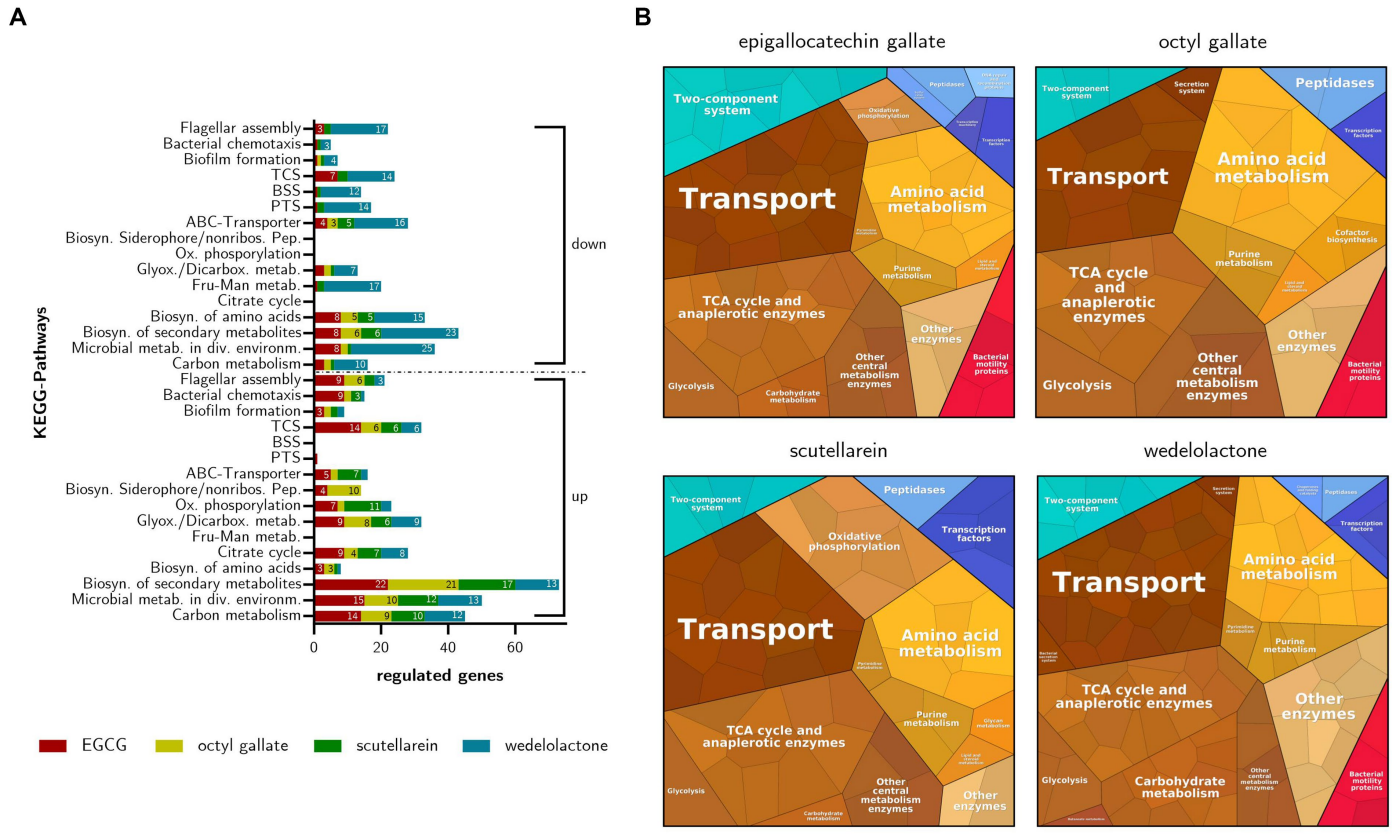
**(A)** Regulated KEGG-Pathways based on differentially expressed genes in inhibited *E. coli* biofilms after treatment with EGCG, octyl gallate, scutellarein or wedelolactone. Influenced pathways are grouped in up- and down-regulation, the depicted numbers represent the number of regulated genes for each treatment. Abbreviated pathway descriptions: Microbial metab. in div. Environm., microbial metabolism in diverse environments; Biosyn. of secondary metabolites, biosynthesis of secondary metabolites; Fru-Man metab., fructose and mannose metabolism; Glyox./Dicarbox. metab., glyoxylate and dicarboxylate metabolism; Ox. phosphorylation, oxidative phosphorylation; Biosyn. Siderophore/nonribos. Pep., biosynthesis of siderophore group non-ribosomal peptides; PTS, phosphotransferase system; BSS, bacterial secretion system; TCS, two-component system. **(B)** Voronoi diagrams of regulated pathways in treated *E. coli* (after 48 h) with four phenolic biofilm inhibitors based on the normalized read counts of differentially expressed genes. Shown are all the significant DEGs (LFC ≥ 1.5, adj. value of *p* ≤ 0.01) irrespective of up- or down-regulation.

In addition to the GO enrichment analysis and in order to obtain a more comprehensive overview of the regulated biological processes involved in the inhibition of biofilm formation in *E. coli*, a KEGG pathway analysis was performed. Overall, the KEGG Mapper revealed that the compounds affected mainly metabolic pathways. The most prevalent pathway was the “biosynthesis of secondary metabolites” with 73 up-regulated and 43 down-regulated genes. Wedelolactone, in particular, showed inhibitory effects. This and the following results are displayed in Figure 8A. In addition to metabolic pathways influenced by all phenolics, other substance-specific effects were observed. EGCG particularly comprehensively regulated the “two-component system” (21 influenced genes ≈ 4.2% of all pathway genes) and the pathways affecting cell motility via “bacterial chemotaxis” (10 ≈ 38%) and “flagellar assembly” (12 ≈ 22%). Octyl gallate appears to influence the “biosynthesis of siderophore group non-ribosomal peptides” (10 ≈ 27%). The regulation of “bacterial chemotaxis” (2 ≈ 7.7%) and “flagellar assembly” (6 ≈ 11%) both play a lesser role. Through scutellarein treatment “oxidative phosphorylation” (11 ≈ 4.9%) as well as “ABC transporters” (12 ≈ 2.3%) are also significantly affected. Comparable to octyl gallate, the regulation of “bacterial chemotaxis” (4 ≈ 15%) and “flagellar assembly” (5 ≈ 9.1%) by scutellarein was rather marginal. Wedelolactone, the compound that had shown the highest number of differentially expressed genes, mainly regulated the “biosynthesis of amino acids” (16 ≈ 6.7%), “fructose and mannose metabolism” (17 ≈ 15%) and “glyoxylate and dicarboxylate metabolism” (16 ≈ 15%). Most of these genes and thus the pathways were negatively affected. The same applies to the “ABC transporters” (17 ≈ 3.3%), the “phosphotransferase system” (14 ≈ 19%), the “bacterial secretion system” (12 ≈ 16%), the “two-component system” (20 ≈ 4.0%) and the “flagellar assembly” (20 ≈ 36%). Only four genes (≈ 15%) were regulated in “bacterial chemotaxis.”

We observed minor influences on the biofilm formation pathway under the influence of the four different compounds (EGCG = 4 [≈ 7%] affected genes, Octyl gallate = 3 [≈5%], Scutellarein = 3 [≈5%], Wedelolactone = 6 [≈10%]). However, octyl gallate inhibited the expression of *csgA*, which is involved in curli-fimbriae biosynthesis, and scutellarein also negatively affected the gene *wza*, which is responsible for colanic acid biosynthesis and transmembrane transport of polysaccharides as components of the extracellular matrix (Collins and Derrick, 2007). *E. coli* can switch from a sessile biofilm-forming lifestyle to a mobile phenotype and several genes have been described as being important in this interplay (Hengge, 2020). All four tested compounds increased the expression of genes involved in the motility of the bacteria, namely the flagellar system. They up-regulated *flhD, flhC* and *fliA* (regulator in the flagellar assembly). In addition, EGCG substantially activated the formation of the basal body and the hook of the flagella. In contrast, wedelolactone led to a reduced expression of 14 genes of the basal body and the hook as well as *flgM, wza* and *pgaB*. A summarized overview of the bacterial processes regulated by the treatments with the natural substances is also given in Figure 8B.

Summing up, in addition to their expected influence on mobility and biofilm formation, the compounds also influence the general metabolism, e.g., amino acid metabolism, glycolysis or TCA cycle and other pathways such as the two-component system or diverse transport systems. The phenolics differ in terms of the number of influenced genes and act differently with respect to some pathways. For example, only scutellarein and EGCG influenced genes of oxidative phosphorylation, and wedelolactone had the most effects on genes belonging to the carbohydrate metabolism and other enzymes.

### 3.7. RT-qPCR

To obtain information on possible temporal changes during the treatment with the compounds, we also performed RT-qPCR analyses of selected genes at early sampling times (12, 24, and 48 h). *CsgD* was chosen because of its well-described function in biofilm formation and the previously demonstrated influence of EGCG (Serra et al., 2016; Hengge, 2019). Also included were genes related to bacterial chemotaxis (*tar* and *motA*) and motility (*fliA, fliC, flhD*), the latter also being described as master regulators in reducing biofilm formation (Lehti et al., 2012).

**Figure 9:**
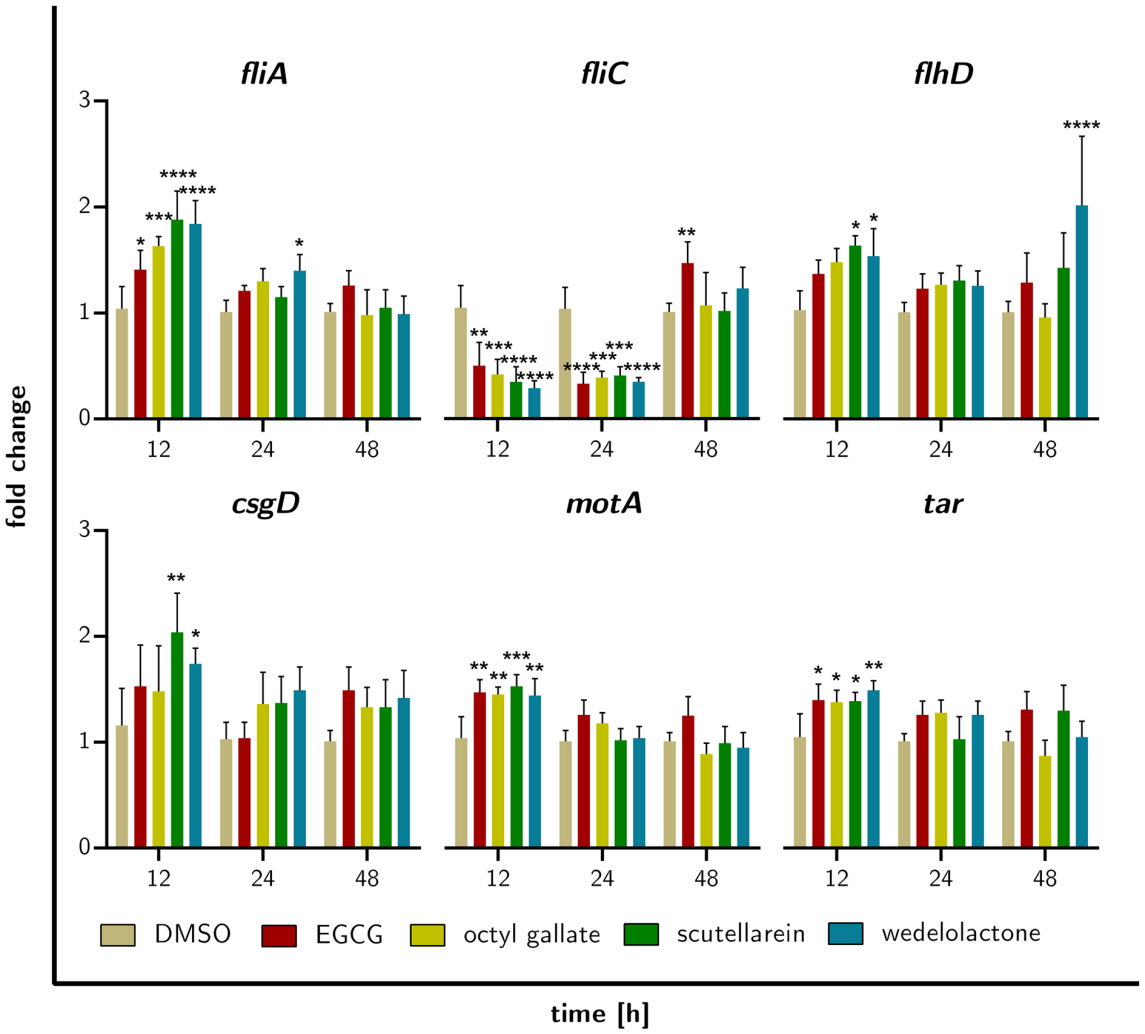
Fold gene expression of the investigated genes that are regulated by treatment of biofilm forming *E. coli* with EGCG, octyl gallate, scutellarein or wedelolactone after incubation for 12, 24, or 48 h. Data obtained with RT-qPCR, statistically tested with GraphPad prism 10.0.1 as two-way ANOVA, Dunnett’s multiple comparisons test (multiple comparison to DMSO), value of ps: *p ≤ 0.05, **p ≤ 0.01, ***p ≤ 0.001, ****p ≤ 0.0001, *ns* > 0.05, mean + SD.

We observed temporal differences as well as compound-specific differences in the expression of the genes analyzed (Figure 9). Overall, most of the genes showed less significant changes in their expression. Nevertheless, after 12 h of incubation, we observed significantly increased expression of *csgD* for scutellarein and a similar trend for the other compounds (not significant). For *fliA,* we observed an up-regulation by octyl gallate, scutellarein and also wedelolactone. *fliC* was significantly down-regulated in response to treatment with these compounds as well as with EGCG. Likewise, *motA, tar* and to a lesser extent *flhD* were mostly significantly up-regulated. After 24 h, *fliA, flhD, motA, tar,* and *csgD* were no longer significantly up-regulated, only the wedelolactone treatment led to such an up-regulation of *fliA*. *FliC* was still significantly down-regulated after 24 h for all treatments. After 48 h no significant changes in the expression of the investigated genes could be observed, except for an up-regulation of *flhD* in response to wedelolactone. However, all four compounds increased the expression of *csgD,* albeit not significantly. Furthermore, *fliC* was now up-regulated for all treatments, with EGCG even causing an increase that reached a level of significance (*p* ≤ 0.007).

### 3.8. Bacterial motility

**Figure 10:**
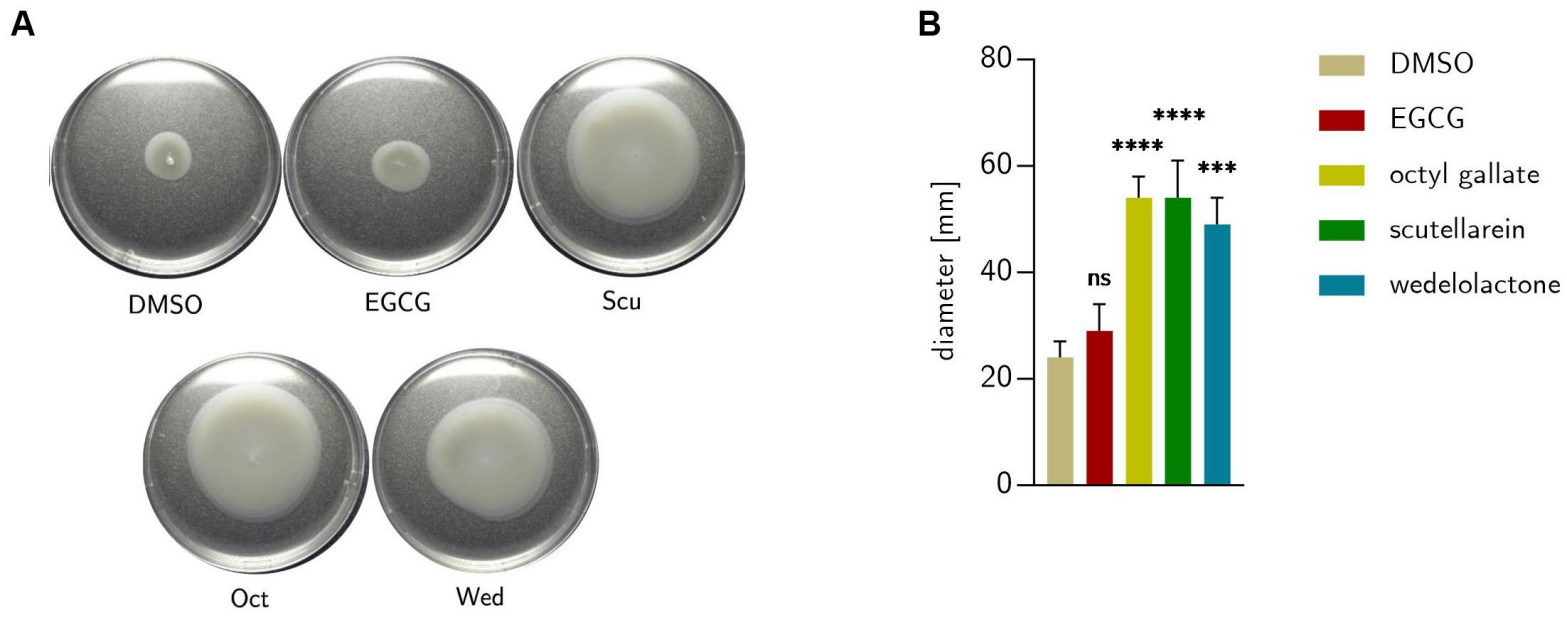
**(A)** Swimming of *E. coli* extracted from treated biofilm colonies formed after 48 h on 0.25% semisolid LB agar after incubation at 28°C for 18 h. **(B)** Motility behavior of the bacteria on semi solid agar compared to untreated DMSO control. Statistical analysis was performed with GraphPad prism 10.0.1 with one-way ANOVA with Dunnett’s multiple comparison. Data represent five independent repetitions. DMSO = dimethyl sulfoxide (control), EGCG, epigallocatechin gallate; Oct, octyl gallate; Scu, scutellarein; Wed, wedelolactone value of ps: *p ≤ 0.05, **p ≤ 0.01, ***p ≤ 0.001, ****p ≤ 0.0001, *ns* > 0.05.

The data obtained from the transcriptome analysis pointed toward altered motility behavior under treatment with phenolic biofilm inhibitors and was therefore explored phenotypically. As Figure 10 shows, wedelolactone, scutellarein and octyl gallate increased bacterial motility, EGCG did not. The following average colony sizes come from three independent measurements: DMSO = 24 ± 3 mm; EGCG = 29 ± 5 mm; Oct = 54 ± 4 mm; Scu = 54 ± 7 mm; Wed = 49 ± 5 mm.

## 4. Discussion

In this study, four structurally different natural phenolic compounds were investigated for their biofilm inhibitory properties against a multidrug-resistant *E. coli* strain of the pandemic lineage ST131. All four tested substances, although structurally unrelated, inhibited macro colony biofilm formation by reducing curli and cellulose production, visible in unstained smooth bacterial colonies compared to the DMSO control (Figure 3A), which was confirmed by the SEM (Figure 5). Consistent with the literature that amyloid fibers such as curli and cellulose play an essential role in biofilm formation, we have seen biofilm structures influenced under treatment: curli and/or fimbriae were obviously reduced. The use of bioimage informatics revealed the same outcome as the visual detection of biofilm inhibition but in a more objective manner (see Supplementary Data S3). A first approach was shown how the post analysis of images of biofilm inhibitors of multidrug-resistant *E. coli* could be achieved.

To gain insights into the pathways that led to a reduction of biofilm formation upon treatment with the compounds, we used an RNA-Seq approach. Subsequently, based on the results, we performed RT-qPCR and bacterial motility experiments. For one of the compounds, EGCG, involvement of the factor *csgD* and a switch toward motility leading to reduced biofilm formation has previously been described in detail (Hengge, 2010; Cui et al., 2012; Serra et al., 2016). In our study, we could not detect a significant regulation of *csgD*. For the other compounds used, namely scutellarein, octyl gallate, and wedelolactone, comprehensive data on their influence on different metabolic pathways in *E. coli* were not yet available. In contrast to other studies (Serra et al., 2016; Pruteanu et al., 2020), the transcriptome of biofilms was analyzed after 48 h of treatment rather than after three or more days, in order to differentially detect the changes in gene expression caused by the different compounds at an early stage. The reason for this procedure was to increase the detection of differentially expressed genes involved in the switch from a sessile to a mobile lifestyle rather than the genes that are highly expressed after this switch.

This experiment was accompanied by RT-qPCR after 12 and 24 h to observe possible time-dependent expression changes for gene regulations of particular interest. As visualized in Figure 8B, overall, a comparable involvement of biological processes was found irrespective of the substance used for treatment, which, keeping in mind the identical phenotype caused by all four compounds, is not a surprising finding. However, our transcriptome analysis also revealed that the different treatments result in few substance-specific changes in gene expression, involving different pathways. Which of these compound-specific changes are actually responsible for biofilm inhibition (alone or in combination) and which are just side effects that are present, is a question that needs to be answered in further studies. Interestingly, the expected strong down-regulation of the well-known regulator *csgD,* which plays an essential role in biofilm formation (Brombacher et al., 2006; Hengge, 2020) was not observable in the data. CsgD activates curli production, a central component of *E. coli* biofilm structures. Upon inhibition, as described for EGCG, biofilm formation is disturbed (Serra et al., 2016; Hengge, 2019). On the contrary, there was a trend toward an up-regulation of *csgD* production when including the RT-qPCR results from the treatment after 12, 24, and 48 h; after 12 h some of the results were even statistically significant, but there was a slight down-regulation of *csgD* under treatment with EGCG and octyl gallate after 48 h of incubation in the RNA-Seq data. Still, the observed up-regulation might reflect an (unsuccessful) attempt by the bacteria to counteract other factors stimulated by the respective treatments. On the other hand, *csgD* up-regulation might be counteracted by post-transcriptional down-regulation, which has been observed for this gene by Holmqvist et al. (2010). This could explain why the treatments we used were still effective even after the observed primary up-regulation of this gene and this might be a worthwhile future research question.

Octyl gallate inhibited the expression of *csgA,* which encodes curlin - the major subunit protein of curli - and is controlled by *csgD* (Barnhart and Chapman, 2006). Wedelolactone had no effect on *csgD* transcription at all and scutellarein enhanced it weakly based on the RNA-Seq and also the RT-qPCR data. In addition, wedelolactone and scutellarein strongly down-regulated *wza*, a gene part of a cluster of genes, that are responsible for the synthesis and the transport of the extracellular polysaccharide colanic acid, which is not relevant for the initial biofilm formation, but for the maturation of biofilms and its deprivation leads to a small and mucoid biofilm (Prigent-Combaret et al., 2000). Wza is a member of the outer membrane auxiliary (OMA) protein family and is required for transportation of biofilm matrix components. Down-regulation of *wza* could therefore be a very plausible explanation for the observed reduction in biofilm formation.

Furthermore, it has been widely described that the motility of bacteria in biofilms is reduced and, conversely, the formation of curli and extracellular matrix components such as colanic acid is switched off in a motile lifestyle (Prigent-Combaret et al., 2000; Hengge, 2019). All examined treatments resulted in an increased expression of genes involved in flagellar motility, e.g., *flhD* and *flhC.* FlhD acts in complex with FlhC as a master transcriptional regulator of several flagellar and non-flagellar operons, including FliA (Barker et al., 2004; Pesavento et al., 2008; Lehti et al., 2012). FliA is a flagellum-specific sigma factor that turns on, e.g., FliC, the basic subunit that polymerizes to form the rigid flagellar filament of *E. coli*, which was also up-regulated. This stepwise regulation could also be observed at the early time points of the RT-qPCR measurements. After 12 and 24 h, *fliC* was down-regulated, but slightly up-regulated after 48 h. *FliC* is controlled by the sigma factor *fliA,* which was already significantly overexpressed after 12 h and still slightly overexpressed after 24 h. As mentioned above, in the case of three of the investigated phenolic substances (octyl gallate, scutellarein, wedelolactone) there was a (partly) strong increase in bacterial motility under treatment (Figure 10) and, thus, a repression of curli formation through counteracting control can be assumed in accordance with the literature (Mika and Hengge, 2013). Although regulation of motility genes was evident, surprisingly, neither upstream regulatory genes of quorum sensing (*qseB, qseC*) (Sperandio et al., 2002) nor other genes responsible for cell–cell communication (Surette et al., 1999; Lima et al., 2023) were affected by treatment conditions.

FliA regulates not only the filaments of the flagella but also their stators. The stator is formed from MotA and MotB and is necessary for the rotation of the flagellar motor (Paul et al., 2010). *MotA* was up-regulated by all substances after 48 h of treatment, most prominently by EGCG and octyl gallate (Figure 7). The results for bacterial motility obtained in the transcriptomic analysis under treatment (especially EGCG and wedelolactone) could be also partly confirmed in the phenotypic motility assays (see Figure 10).

In line with the detected up-regulation of motility-related genes and the phenotypic specification, genes related to chemotaxis like *cheY* or *tar* were also influenced under treatment as chemotaxis enables bacteria to move specifically to avoid harmful environmental influences, e.g., xenobiotics. CheY is involved in the transmission of sensory signals from the chemoreceptors to the flagellar motors and its overexpression in association with MotA and MotB improves motility (Paul et al., 2010). *CheY* is regulated via some intermediate steps by membrane receptors of the methyl-accepting chemotaxis protein complex (MCPs) like Tar (Kanehisa and Sato, 2020). We observed an up-regulation of *tar* by all four compounds, which makes sense as Tar has been described as target for phenol before (Imae et al., 1987).

Moreover some genes important for fimbriae production were downregulated, particularly by the wedelolactone treatment. The genes belong to the fimbrial usher porin family *fimC, fimD, fimF* and *fimG* (Busch and Waksman, 2012) as well as the chaperone-usher fimbrial operons *yadV, yehC, ydeQ,* and *ydeS* (Wurpel et al., 2013). EGCG down regulated three of them (*yadV, ydeQ,* and *ydeS*) and scutellarein two (*ydeQ* and *fimD*). As mentioned above, octyl gallate inhibited the expression of *csgA* (coding for a subunit protein of curli) that could result in the reduced biofilm formation. These findings could explain the apparently reduced filamentous network of the biofilms in the scanning electron micrographs (Figure 5). In accordance with the literature the remained structures are assumable flagella (Friedlander et al., 2013; Serra et al., 2013; Kleta et al., 2014), which is also reasonable based on the increased expression of motility genes.

Aside from the complex interplay of biofilm formation and motility, diverse metabolic pathways have also been described as being linked to biofilm formation (Al Mamun et al., 2012). We found that genes involved in arginine biosynthesis (*argA, argB, argC,* and *argD*) and uptake (genes for arginine:ornithine antiporter YdgI and ABC-transporter ArtJ) were also down-regulated under treatment. These observations are in line with recent observations by Lu et al. (2019). Who reported extensive metabolic reprogramming through metabolic pathways including arginine biosynthesis and the connected TCA-cycle that trigger the biofilm formation in uropathogenic *E. coli* (Lu et al., 2019). Recently it has also been shown that arginine acts as a metabolic precursor that promotes biofilm formation in *Pseudomonas aeruginosa* (Liu et al., 2022).

## 5. Conclusion

The comparative analysis of the pathways influencing biofilm formation addressed by four structurally different phenolic compounds, namely EGCG, octyl gallate, scutellarein and wedelolactone, revealed that all compounds, irrespective of their structure, mainly influenced the same pathways. As expected, these pathways included bacterial motility, chemotaxis, biofilm formation but also metabolic processes such as amino acid metabolism and the tricarboxylic acid cycle. The scope of the influence of the treatments did however vary. Whether the observed metabolic changes are primarily the cause of the reduced biofilm formation or rather the result of the switch of the bacteria from a sessile to a mobile lifestyle remains to be elucidated by further studies.

## Data availability statement

The datasets presented in this study can be found in online repositories. The names of the repository/repositories and accession number(s) can be found at: https://www.ebi.ac.uk/ena, PRJEB62597.

## Author contributions

SG, DB, and NS planned the concept of this study. DB conducted the experiments and did most of the bioinformatical as well as the statistical analyses with the help of MS. RS performed the SEM. RW and SR performed the bioimage analyses. SG was responsible for the administration. All authors contributed to manuscript revision, read, and approved the submitted version.

## Funding

This research was partly funded by the German Federal Ministry of Education and Research (BMBF), grant number 01KI2015 (DISPATch_MRGN) to KS. We also acknowledge financial support by University of Greifswald within the funding program Open Access Publikationsfonds.

## Conflict of interest

The authors declare that the research was conducted in the absence of any commercial or financial relationships that could be construed as a potential conflict of interest.

## Publisher’s note

All claims expressed in this article are solely those of the authors and do not necessarily represent those of their affiliated organizations, or those of the publisher, the editors and the reviewers. Any product that may be evaluated in this article, or claim that may be made by its manufacturer, is not guaranteed or endorsed by the publisher.
