# Supplementary material for: Natural phenolic compounds as biofilm inhibitors of multidrug-resistant Escherichia coli – the role of similar biological processes despite structural diversity
Source: Front Microbiol. 2023 Sep 4;14:1232039. doi: 10.3389/fmicb.2023.1232039 (PMC10507321; doi:10.3389/fmicb.2023.1232039)
Supplement: Supplementary file 1 [file Table_1.docx]

Supplementary Table 1. Primers used in this study and their functionality.

| **gene** | **Protein name** | | **primer (5’-3’)** | **functionality** |
| --- | --- | --- | --- | --- |
| *tar* | | methyl-accepting chemotaxis protein II | Forward: GCGGCGGGCAACACCGATCTTTCCTCC  Reverse: GCGGTGTCGGAGGCACTTTGCGCC | chemotaxis |
| *csgD* | | CsgBAC operon transcriptional regulatory protein | Forward:  TGCAAGGCGTCCTGCGCGGCG  Reverse: CGCGCCGATACGCAGCTTATTCAGG | biofilm formation |
| *fliA* | | RNA polymerase sigma factor FliA | Forward: GCAACGCCACGGAAACTGAGGTAGCGG  Reverse: CGTGCTCTTCGCGCCACTCATCGTAGG | flagellar assembly |
| *flhD* | | flagellar transcriptional regulator FlhD | Forward: ACAACGTTAGCGGCACTGACTCTTCCGC  Reverse:  CAGGCCCTTTTCTTGCGCAGCG | flagellar assembly |
| *fliC* | | flagellin | Forward: TGCTGCGGATGTCAAGGATGCTGGGG  Reverse: CTGCACCAACAGTTACCGCCGCCGC | flagellar assembly |
| *motA* | | motility protein A | Forward: GCGGGCGGGCAATTAGTGGCTATCTGG  Reverse: GCTGACGCTGAAACTCCGGCCCGAG | chemotaxis |
| *rrsA* | | 16s-RNA | Forward: CCCGCACAAGCGGTGGAGCATGTGG  Reverse: CGGCCGGACCGCTGGCAACAAAGG | Transcription (reference) |
| *rpoA* | | DNA-directed RNA polymerase subunit alpha | Forward: CAGCGCGGTCGTGGTTATGTGCCGG  Reverse: GGCCGATTGGGCGCTCATCTTCTTCCG | Transcription (reference) |
| *dnaE* | | DNA polymerase III subunit alpha | Forward: CCTGACGGTGCTGGCGGCGAACAATACC  Reverse: GCGCCCGCCGGAAAGAAGGATCAACCC | DNA replication (reference) |
