# Supplementary material for: Natural phenolic compounds as biofilm inhibitors of multidrug-resistant Escherichia coli – the role of similar biological processes despite structural diversity
Source: Front Microbiol. 2023 Sep 4;14:1232039. doi: 10.3389/fmicb.2023.1232039 (PMC10507321; doi:10.3389/fmicb.2023.1232039)
Supplement: Supplementary file 2 [file Table_2.docx]

SUPPLEMENTARY TABLE 2. RNA content and read counts of *E. coli* biofilm colonies after treatment with five conditions (dimethylsulfoxide [DMSO = solvent control], epigallocatechin gallate [EGCG], octyl gallate [Oct], scutellarein [Scu] and wedelolactone [Wed]). The biological replicates are indicated through indexed numbers.

| **Sample** | **RNA content [ng/µL]** | **RAW reads [10^6^]** | **Cleaned reads [10^6^]** |
| --- | --- | --- | --- |
| DMSO_1 | 1761.4 | 15.3 | 12.4 |
| DMSO_2 | 2126.8 | 16.7 | 15.4 |
| EGCG_1 | 992.2 | 17.1 | 14.8 |
| EGCG_2 | 712.9 | 16.3 | 14.8 |
| Oct_1 | 853.5 | 16.7 | 14.8 |
| Oct_2 | 883.8 | 18.0 | 15.5 |
| Scu_1 | 1851.7 | 14.5 | 12.8 |
| Scu_2 | 992.2 | 15.0 | 13.0 |
| Wed_1 | 1353.7 | 14.7 | 13.0 |
| Wed_2 | 1182.2 | 16.5 | 14.7 |
