# Supplementary material for: Natural phenolic compounds as biofilm inhibitors of multidrug-resistant Escherichia coli – the role of similar biological processes despite structural diversity
Source: Front Microbiol. 2023 Sep 4;14:1232039. doi: 10.3389/fmicb.2023.1232039 (PMC10507321; doi:10.3389/fmicb.2023.1232039)
Supplement: Supplementary file 4 [file Data_Sheet_2.docx]

SUPPLEMENTARY DATA 1. FastQ reports of Samples. P01-A01-1-3D = DMSO_1; P01-F01-1-4D = DMSO_2; P01-B01-2-3E = EGCG_1; P01-G01-2-4E = EGCG_2; P01-C01-3-3O = Oct_1; P01-H01-3-4O = Oct_2; P01-D01-4-3S = Scu_1; P01-A02-4-4S = Scu_2; P01-E01-5-3W = Wed_1; P01-B02-5-4W = Wed_2.
