# Supplementary material for: Natural phenolic compounds as biofilm inhibitors of multidrug-resistant Escherichia coli – the role of similar biological processes despite structural diversity
Source: Front Microbiol. 2023 Sep 4;14:1232039. doi: 10.3389/fmicb.2023.1232039 (PMC10507321; doi:10.3389/fmicb.2023.1232039)
Supplement: Supplementary file 5 [file Data_Sheet_3.pdf]

Analysis of biofilm inhibitors of multidrug-resistant *E. coli*

David Buchmann* Michael Schwabe† Romano Weiss‡ Andreas W. Kuss§
Katharina Schaufler¶ Rabea Schlüter‖ Stefan Rödiger** Sebastian Guenther††
Nadin Schultze‡‡

05 August, 2023

Contents

0.1 Read the raw data 2

1 Introduction **2**

2 Methods and Materials **4**

3 Analysis and Results **4**
3.1 Processing of the imported data 4
3.2 Visualitzation of the dose-response relationship 5

4 Discussion and Conclusion **6**

5 Data dump form the import data **7**

References **14**

*Pharmaceutical Biology, Institute of Pharmacy, University of Greifswald, Germany
†Pharmaceutical Microbiology, Institute of Pharmacy, University of Greifswald, Germany
‡Institute of Biotechnology, Faculty Environment and Natural Sciences, Brandenburg University of Technology Cottbus-Senftenberg, Germany
§Department of Functional Genomics, Interfaculty Institute of Genetics and Functional Genomics, University of Greifswald, Germany
¶Pharmaceutical Microbiology, Institute of Pharmacy, University of Greifswald, Germany. Institute of Infection Medicine, Christian-Albrecht University Kiel and University Medical Center Schleswig-Holstein, Germany
‖Imaging Center of the Department of Biology, University of Greifswald, Germany
**Institute of Biotechnology, Faculty Environment and Natural Sciences, Brandenburg University of Technology Cottbus-Senftenberg, Germany, stefan.roediger@b-tu.de
††Pharmaceutical Biology, Institute of Pharmacy, University of Greifswald, Germany
‡‡Pharmaceutical Biology, Institute of Pharmacy, University of Greifswald, Germany, Corresponding Author nadin.schultze@uni-greifswald.de

Packages for data import and processing:

0.1 Read the raw data

Import of the data via the `import()` function of the `rio` package.

1 Introduction

This document deals with the objectified, reproducible analysis of biofilm inhibitors of multidrug-resistant *E. coli* using bioimage informatics. Bioimage informatics can be used to obtain quantitative information from image data (Schneider et al. 2019). Our experiments strongly suggested that the controls differed from the treated samples in their surface area (see *Figure 4 main text*). The controls where marked growth is observed have a highly textured surface. In contrast, samples in which growth is influenced by the test substance tend to have a smooth surface. In addition, concentration-dependent gray levels can be observed. We have taken these as the basis for a first algorithm as exemplified in Figure 1 below. This algorithm determines from all image data statis parameters that describe the intensity (mean) and surface condition (standard deviation).

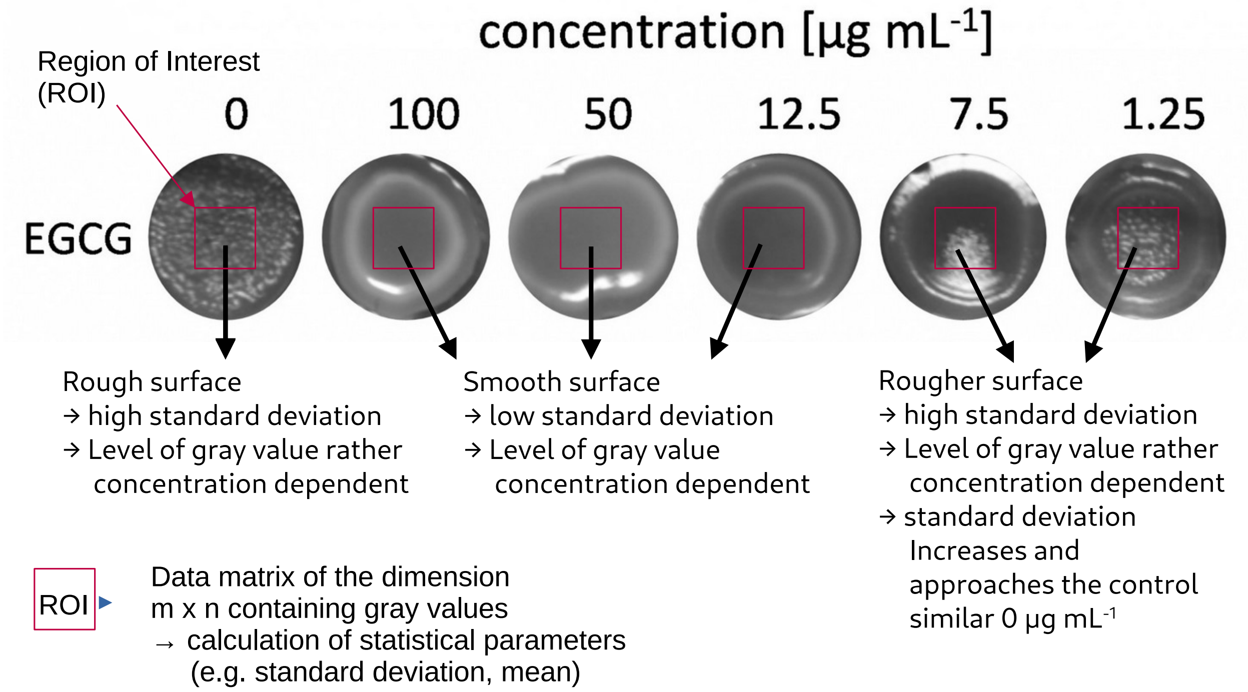

Figure 1: The standard deviation as a parameter for the classification of biofilms. Our analysis of the raw data, showed that the standard deviation within the ROI is a reliable parameter to distinguish between intact biofilm and inhibited biofilms. Intact biofilms (Bf0, samples with very low inhibitor concentration) have a high standard deviation. Controls (agar) and samples with high inhibitor concentration have a low standard deviation.

To be able to evaluate the data in a concentration-dependent manner, it has been shown that the standard deviation is a good threshold parameter for estimating whether a biofilm can form in a well (Figure 2). In general, biofilms have a high standard deviation and high intensity value. Wells where a substance influences the growth have a low standard deviation. The effect of a substance correlates with the intensity of the gray levels.

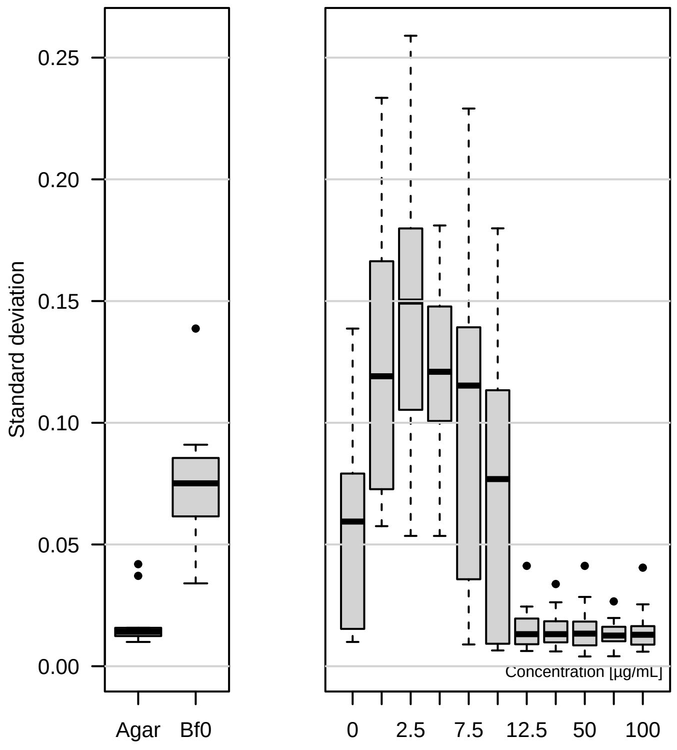

Figure 2: The standard deviation as a parameter for the classification of biofilms. Our analysis of the raw data, showed that the standard deviation within the ROI is a reliable parameter to distinguish between intact biofilm and inhibited biofilms. Intact biofilms (Bf0, samples with very low inhibitor concentration) have a high standard deviation. Controls (agar) and samples with high inhibitor concentration have a low standard deviation. Left: Standard deviation of Agar (no biofilm, smooth surface) and Standard deviation of Bf0 (non-inhibited biofilm, rough biofilm). Right: Standard deviation of all tested substances (EGCG (EGCG), Octyl gallate (Oct), Scutellarein (Scu) and Wedelolactone (Wed)) at the corresponding inhibitor concentration.

2 Methods and Materials

The R programming language (v. 4.3.1) was used to analyze the image data using the RKWard (v. 0.7.5z+0.7.6+devel1) programming environment (Rödiger et al. 2012). The `imager` package (Schneider et al. 2019) was used to obtain the data matrices from the images. R scripts were created for all steps. In broad strokes, the image data was analyzed as follows.

1. import of the image data using the `load.image()` function
2. conversion of the color image into an 8 bit grayscale image using the `grayscale()` function.
3. since the images were taken at different distances, illumination and perspectives, an adjustments were necessary. We noticed that the inter-plate illumination variance is the main cause the variances. For this purpose, the mean brightness of each image was determined. The global mean value was calculated from the total of all mean image brightnesses. The correction value for the corresponding brightness adjustment for each image was the distance from the mean image brightness to the global image brightness.
4. regions of interest (ROI) were interactively determined from each biofilm using the in-house developed `roi()` function (file: `roi.R`). The function uses the `grabRect()` function from the `imager` package to calculate rectangular areas from the images. The coordinates are stored in the file `biofilm_roi_data.ods`.
5. using the previously determined coordinates, the specially developed `wizart()` function was used to determine the gray values from the ROIs (file: `wizart.R`). The function uses the `imsub()` function of the `imager` package. Statistical parameters (mean, standard deviation) were determined from these image areas.

3 Analysis and Results

3.1 Processing of the imported data

Table 1: Aggregated data from imported raw data. substances: EGCG, EGCG; Oct, Octyl gallate; Scu, Scutellarein; Wed, Wedelolactone; Agar: argar negative control; Bf0: DMSO control. conc: concentration in µg/mL. average: mean of n values. dispersion: standard deviation of n values. n: number of ROIs used for the analysis. thereshold: standard deviation of the control ROIs.

substances	conc	average	dispersion	n	threshold
Agar	0.00	0.3000932	0.0820711	9	0.0190357
Bf0	0.00	0.3890785	0.0562580	15	0.0748746
EGCG	100.00	0.4310053	0.0710799	5	0.0181656
EGCG	75.00	0.4483073	0.0517782	5	0.0161081
EGCG	50.00	0.4417229	0.0525739	5	0.0172100
EGCG	25.00	0.3941308	0.0574706	5	0.0160132
EGCG	12.50	0.3546563	0.0557085	5	0.0150549
EGCG	10.00	0.2529716	0.0154291	3	0.0471066
EGCG	7.50	0.4866774	0.0755143	3	0.1815289
EGCG	5.00	0.4925925	0.1143001	3	0.1313111
EGCG	2.50	0.4957951	0.1145032	3	0.1735204
EGCG	1.25	0.3791415	0.1040813	3	0.1621736
Oct	100.00	0.4116602	0.0409751	5	0.0104746
Oct	75.00	0.4365857	0.0554384	5	0.0105795
Oct	50.00	0.4417308	0.0439093	5	0.0125659
Oct	25.00	0.4317778	0.0483656	5	0.0133327
Oct	12.50	0.3423661	0.0672765	5	0.0166867
Oct	10.00	0.2243703	0.0143602	3	0.0423025

substances	conc	average	dispersion	n	threshold
Oct	7.50	0.2310631	0.0201285	3	0.0121551
Oct	5.00	0.4531742	0.1404303	3	0.1472398
Oct	2.50	0.4886121	0.0571704	3	0.1696486
Oct	1.25	0.4040173	0.0583455	3	0.1488059
Scu	100.00	0.4536837	0.0868236	5	0.0177507
Scu	75.00	0.4510484	0.0866292	5	0.0147664
Scu	50.00	0.4284131	0.0911348	5	0.0106111
Scu	25.00	0.3612994	0.1070053	5	0.0111493
Scu	12.50	0.3116210	0.0754736	5	0.0144313
Scu	10.00	0.3946905	0.0233637	3	0.1129153
Scu	7.50	0.4276121	0.0254355	3	0.1059458
Scu	5.00	0.3883310	0.0950384	3	0.1048288
Scu	2.50	0.3919454	0.0962755	3	0.1201654
Scu	1.25	0.3608539	0.0583200	3	0.0889731
Wed	100.00	0.4442955	0.0821965	5	0.0105854
Wed	75.00	0.4093229	0.0948942	5	0.0120229
Wed	50.00	0.3785229	0.0982235	5	0.0186566
Wed	25.00	0.3363426	0.1044078	5	0.0184151
Wed	12.50	0.3050327	0.0810679	5	0.0141415
Wed	10.00	0.3232069	0.0474933	3	0.0902261
Wed	7.50	0.3357689	0.0420405	3	0.0961608
Wed	5.00	0.3512853	0.0754461	3	0.0967966
Wed	2.50	0.3502003	0.0660119	3	0.1138771
Wed	1.25	0.3004029	0.0538019	3	0.1056180

3.2 Visualitzation of the dose-response relationship

The following plot show lineplots of the mean and standard error of a response variable for one-way experimental design for the substances EGCG (EGCG), Octyl gallate (Oct), Scutellarein (Scu) and Wedelolactone (Wed). Explanation of the data presented. In the plots, one can see a dose-dependent increase in intensity. The intensity is a measure of the gray levels. High values correspond to areas that are almost white, i.e. where no biofilm can be detected. Low values correspond to areas that tend to lower gray levels. These are areas where biofilms are forming. Thus, the interval shown here represents the range in which inhibition of the biofilm is measurable. No values are given in the ranges of low concentrations of the substances, as these are removed due to the automatic filtering algorithm.

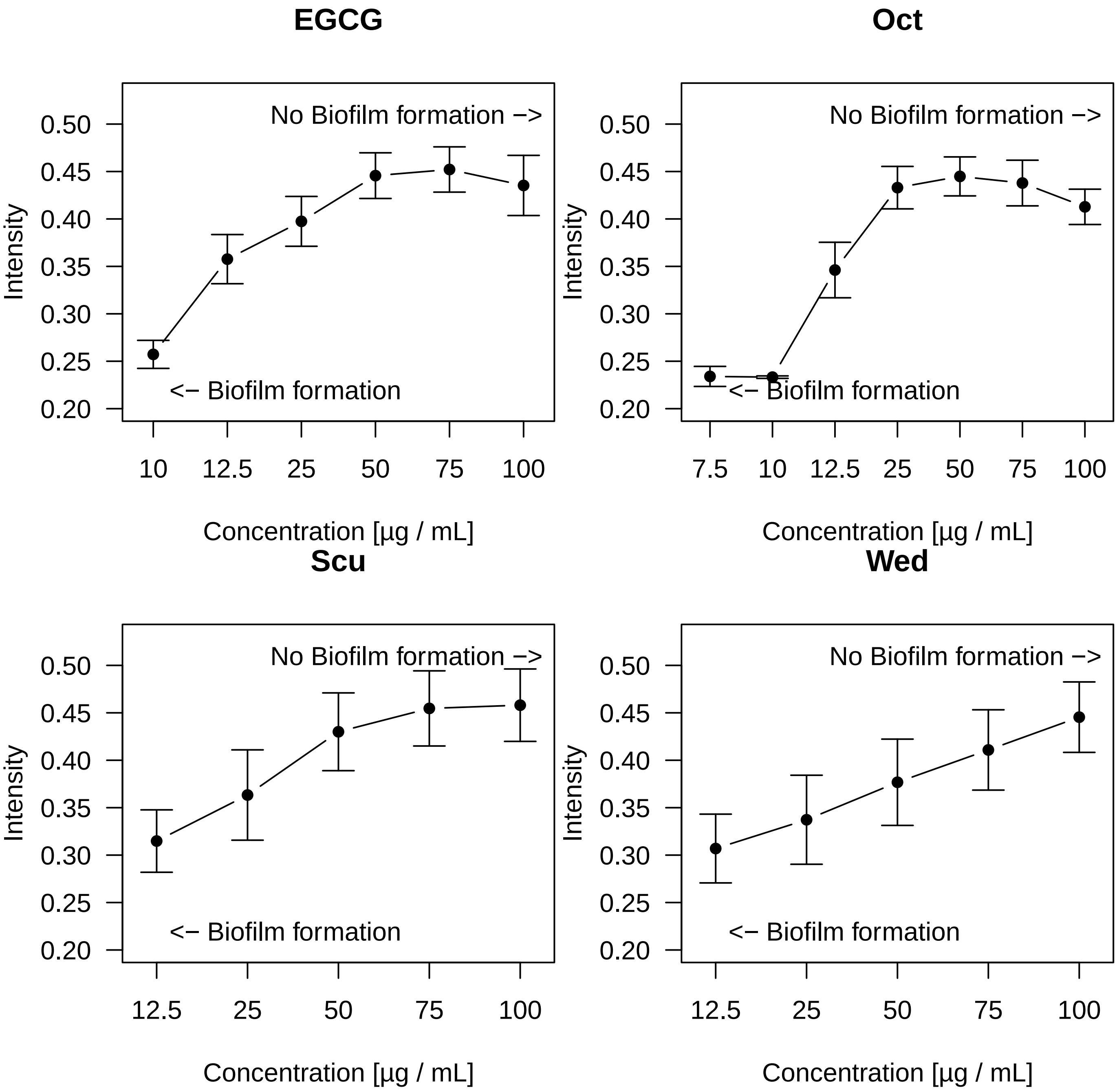

4 Discussion and Conclusion

In this document, the first approach was shown how the analysis of biofilm inhibitors of multidrug-resistant *E. coli* could be achieved by more objective machine methods. The approach we have chosen objectifies the analysis and compensates for inter-plate variances with respect to brightness. In future work, we will further optimize the system, as interference did not allow full automation. We plan to address the following aspects in the future.

- Since not all plates were taken with the same spacing and position, we were not able to implement an algorithm that would automatically detect each well with reasonable effort.
- The uneven illumination with a point or rod-shaped light source resulted in reflections and shadows in

the wells. These could also not be accounted for with reasonable effort here. In future work we plan to use LED Ring Light Source from the Monocular / Stereo Microscope.

- Currently, only intensity and scattering parameters are recorded. In future analyses, we will also determine geometric information, such as the shape factor or diameter, in order to create multiparametric models from the data.
- The current software requires knowledge of the programming language R, bioimage informatics and the handling of different data structures. These skills usually cannot be expected from every scientist. Therefore, we want to extend the software and add a graphical user interface.

5 Data dump form the import data

Table 2: Data of the imported raw data. image_name: file name of the analyzed image. substances: EGCG, EGCG; Oct, Octyl gallate; Scu, Scutellarein; Wed, Wedelolactone; Agar: argar negative control; Bf0: DMSO control. conc: concentration in µg/mL. mean: mean intensity of ROI per image. sd: standard deviation intensity of ROI per image. median: median intensity of ROI per image. IQR: inter quartile range of ROI per image.

image_name	substances	conc	mean	sd	median	IQR
EGCG_Oct_Scu_Wed_MBIC_Vers1_100 12.5.PNG	Agar	0.00	0.4923235	0.0371157	0.4913168	0.0543922
EGCG_Oct_Scu_Wed_MBIC_Vers2_100 12.5.PNG	Agar	0.00	0.3483981	0.0419149	0.3574760	0.0653431
EGCG_Oct_Scu_Wed_MBIC_Vers3_100 12.5mg.PNG	Agar	0.00	0.3193608	0.0099639	0.3163897	0.0153333
EGCG_Oct_Scu_Wed_MBIC_Vers3_100 1.25.PNG	Agar	0.00	0.2621936	0.0148805	0.2645644	0.0240392
EGCG_Oct_Scu_Wed_MBIC_Vers3_100 1.25.PNG	Agar	0.00	0.2340175	0.0141822	0.2320938	0.0205490
EGCG_Oct_Scu_Wed_MBIC_Vers4_100 12.5.PNG	Agar	0.00	0.2873984	0.0157705	0.2884796	0.0233333
EGCG_Oct_Scu_Wed_MBIC_Vers4_1- 1.25_Reihe2.PNG	Agar	0.00	0.2485785	0.0136156	0.2499810	0.0201569
EGCG_Oct_Scu_Wed_MBIC_Vers5_100 12.5_Reihe2.PNG	Agar	0.00	0.2371938	0.0115011	0.2387957	0.0098431
EGCG_Oct_Scu_Wed_MBIC_Vers5_1- 1.25_Reihe2.PNG	Agar	0.00	0.2623178	0.0123772	0.2617419	0.0199608
EGCG_Oct_Scu_Wed_MBIC_Vers1_100 12.5.PNG	Bf0	0.00	0.5466425	0.1386986	0.4946110	0.1483333
EGCG_Oct_Scu_Wed_MBIC_Vers1_100 12.5.PNG	Bf0	0.00	0.4755765	0.0909908	0.4471992	0.0827941
EGCG_Oct_Scu_Wed_MBIC_Vers1_100 12.5.PNG	Bf0	0.00	0.4723527	0.0751292	0.4543365	0.0683137
EGCG_Oct_Scu_Wed_MBIC_Vers2_100 12.5.PNG	Bf0	0.00	0.4068420	0.0823446	0.3868486	0.1001471
EGCG_Oct_Scu_Wed_MBIC_Vers2_100 12.5.PNG	Bf0	0.00	0.3849689	0.0871041	0.3614368	0.0937647
EGCG_Oct_Scu_Wed_MBIC_Vers2_100 12.5.PNG	Bf0	0.00	0.3479253	0.0839776	0.3278682	0.0839216
EGCG_Oct_Scu_Wed_MBIC_Vers3_100 12.5mg.PNG	Bf0	0.00	0.4058418	0.0340356	0.3990956	0.0448235

image_name	substances	conc	mean	sd	median	IQR
EGCG_Oct_Scu_Wed_MBIC_Vers3_100 12.5mg.PNG	Bf0	0.00	0.4297384	0.0458486	0.4244682	0.0626275
EGCG_Oct_Scu_Wed_MBIC_Vers3_100 12.5mg.PNG	Bf0	0.00	0.4484685	0.0606164	0.4416839	0.1078137
EGCG_Oct_Scu_Wed_MBIC_Vers4_100 12.5.PNG	Bf0	0.00	0.3839049	0.0664974	0.3717541	0.0829804
EGCG_Oct_Scu_Wed_MBIC_Vers4_100 12.5.PNG	Bf0	0.00	0.3943848	0.0759300	0.3803227	0.1052353
EGCG_Oct_Scu_Wed_MBIC_Vers4_100 12.5.PNG	Bf0	0.00	0.2943619	0.0874420	0.2770286	0.1091961
EGCG_Oct_Scu_Wed_MBIC_Vers5_100 12.5_Reihe2.PNG	Bf0	0.00	0.3903372	0.0624685	0.3789525	0.0714510
EGCG_Oct_Scu_Wed_MBIC_Vers5_100 12.5_Reihe2.PNG	Bf0	0.00	0.3525204	0.0738161	0.3414231	0.0848235
EGCG_Oct_Scu_Wed_MBIC_Vers5_100 12.5_Reihe2.PNG	Bf0	0.00	0.3580319	0.0582187	0.3491486	0.0682745
EGCG_Oct_Scu_Wed_MBIC_Vers1_100 12.5.PNG	EGCG	100.00	0.5544704	0.0154822	0.5526894	0.0221373
EGCG_Oct_Scu_Wed_MBIC_Vers1_100 12.5.PNG	EGCG	75.00	0.5272235	0.0198014	0.5218659	0.0274314
EGCG_Oct_Scu_Wed_MBIC_Vers1_100 12.5.PNG	EGCG	50.00	0.5139101	0.0183465	0.5095914	0.0147843
EGCG_Oct_Scu_Wed_MBIC_Vers1_100 12.5.PNG	EGCG	25.00	0.4815097	0.0179162	0.4788070	0.0190588
EGCG_Oct_Scu_Wed_MBIC_Vers1_100 12.5.PNG	EGCG	12.50	0.4362450	0.0144021	0.4324541	0.0173431
EGCG_Oct_Scu_Wed_MBIC_Vers2_100 12.5.PNG	EGCG	100.00	0.4035046	0.0171265	0.3982799	0.0180392
EGCG_Oct_Scu_Wed_MBIC_Vers2_100 12.5.PNG	EGCG	75.00	0.4553443	0.0132399	0.4527309	0.0171373
EGCG_Oct_Scu_Wed_MBIC_Vers2_100 12.5.PNG	EGCG	50.00	0.4420443	0.0186501	0.4367309	0.0311765
EGCG_Oct_Scu_Wed_MBIC_Vers2_100 12.5.PNG	EGCG	25.00	0.3514486	0.0207695	0.3464564	0.0210588
EGCG_Oct_Scu_Wed_MBIC_Vers2_100 12.5.PNG	EGCG	12.50	0.3284989	0.0205887	0.3273191	0.0276863
EGCG_Oct_Scu_Wed_MBIC_Vers3_100 12.5mg.PNG	EGCG	100.00	0.4109394	0.0086971	0.4098407	0.0117647
EGCG_Oct_Scu_Wed_MBIC_Vers3_100 12.5mg.PNG	EGCG	75.00	0.4394269	0.0118414	0.4374290	0.0156863
EGCG_Oct_Scu_Wed_MBIC_Vers3_100 12.5mg.PNG	EGCG	50.00	0.4272812	0.0136246	0.4241152	0.0168235
EGCG_Oct_Scu_Wed_MBIC_Vers3_100 12.5mg.PNG	EGCG	25.00	0.3715286	0.0078567	0.3706250	0.0108039
EGCG_Oct_Scu_Wed_MBIC_Vers3_100 12.5mg.PNG	EGCG	12.50	0.3331080	0.0087093	0.3306250	0.0125882
EGCG_Oct_Scu_Wed_MBIC_Vers3_100 1.25.PNG	EGCG	10.00	0.3055156	0.1257317	0.2466036	0.0652745
EGCG_Oct_Scu_Wed_MBIC_Vers3_100 1.25.PNG	EGCG	7.50	0.5250372	0.1385938	0.5097801	0.2099608
EGCG_Oct_Scu_Wed_MBIC_Vers3_100 1.25.PNG	EGCG	5.00	0.5677411	0.1227029	0.5611526	0.1810784

image_name	substances	conc	mean	sd	median	IQR
EGCG_Oct_Scu_Wed_MBIC_Vers3_100-1.25.PNG	EGCG	2.50	0.5947637	0.1603931	0.5738977	0.2579314
EGCG_Oct_Scu_Wed_MBIC_Vers3_100-1.25.PNG	EGCG	1.25	0.5234614	0.1423735	0.4982899	0.1953725
EGCG_Oct_Scu_Wed_MBIC_Vers4_100-12.5.PNG	EGCG	100.00	0.4377611	0.0404835	0.4252639	0.0512157
EGCG_Oct_Scu_Wed_MBIC_Vers4_100-12.5.PNG	EGCG	75.00	0.4606295	0.0266117	0.4529894	0.0318824
EGCG_Oct_Scu_Wed_MBIC_Vers4_100-12.5.PNG	EGCG	50.00	0.4749814	0.0284561	0.4695384	0.0369608
EGCG_Oct_Scu_Wed_MBIC_Vers4_100-12.5.PNG	EGCG	25.00	0.4353948	0.0262643	0.4269110	0.0341569
EGCG_Oct_Scu_Wed_MBIC_Vers4_100-12.5.PNG	EGCG	12.50	0.3974925	0.0245076	0.3898521	0.0245098
EGCG_Oct_Scu_Wed_MBIC_Vers4_1-1.25_Reihe2.PNG	EGCG	10.00	0.2424603	0.0077724	0.2417457	0.0092941
EGCG_Oct_Scu_Wed_MBIC_Vers4_1-1.25_Reihe2.PNG	EGCG	7.50	0.5279508	0.1769088	0.5479417	0.3047059
EGCG_Oct_Scu_Wed_MBIC_Vers4_1-1.25_Reihe2.PNG	EGCG	5.00	0.5460512	0.1438032	0.5559810	0.2153431
EGCG_Oct_Scu_Wed_MBIC_Vers4_1-1.25_Reihe2.PNG	EGCG	2.50	0.3937466	0.1011821	0.3643535	0.1329706
EGCG_Oct_Scu_Wed_MBIC_Vers4_1-1.25_Reihe2.PNG	EGCG	1.25	0.3437890	0.1106687	0.3059417	0.0444314
EGCG_Oct_Scu_Wed_MBIC_Vers5_100-12.5_Reihe2.PNG	EGCG	100.00	0.3698381	0.0090389	0.3689525	0.0122745
EGCG_Oct_Scu_Wed_MBIC_Vers5_100-12.5_Reihe2.PNG	EGCG	75.00	0.3780118	0.0090460	0.3765212	0.0117255
EGCG_Oct_Scu_Wed_MBIC_Vers5_100-12.5_Reihe2.PNG	EGCG	50.00	0.3700201	0.0069726	0.3686388	0.0102745
EGCG_Oct_Scu_Wed_MBIC_Vers5_100-12.5_Reihe2.PNG	EGCG	25.00	0.3474119	0.0072594	0.3478545	0.0100392
EGCG_Oct_Scu_Wed_MBIC_Vers5_100-12.5_Reihe2.PNG	EGCG	12.50	0.2927980	0.0070669	0.2930310	0.0089412
EGCG_Oct_Scu_Wed_MBIC_Vers5_1-1.25_Reihe2.PNG	EGCG	10.00	0.2719987	0.0078158	0.2705654	0.0106667
EGCG_Oct_Scu_Wed_MBIC_Vers5_1-1.25_Reihe2.PNG	EGCG	7.50	0.5152016	0.2290843	0.4023105	0.4003824
EGCG_Oct_Scu_Wed_MBIC_Vers5_1-1.25_Reihe2.PNG	EGCG	5.00	0.4098904	0.1274272	0.3606438	0.1292255
EGCG_Oct_Scu_Wed_MBIC_Vers5_1-1.25_Reihe2.PNG	EGCG	2.50	0.6059437	0.2589858	0.5491340	0.5803922
EGCG_Oct_Scu_Wed_MBIC_Vers5_1-1.25_Reihe2.PNG	EGCG	1.25	0.4662011	0.2334787	0.3331929	0.3505294
EGCG_Oct_Scu_Wed_MBIC_Vers1_100-12.5.PNG	Oct	100.00	0.4856701	0.0100818	0.4832776	0.0156863
EGCG_Oct_Scu_Wed_MBIC_Vers1_100-12.5.PNG	Oct	75.00	0.5316216	0.0119280	0.5331600	0.0187843
EGCG_Oct_Scu_Wed_MBIC_Vers1_100-12.5.PNG	Oct	50.00	0.5194401	0.0160091	0.5139051	0.0223922
EGCG_Oct_Scu_Wed_MBIC_Vers1_100-12.5.PNG	Oct	25.00	0.5040200	0.0126525	0.5017090	0.0147059

image_name	substances	conc	mean	sd	median	IQR
EGCG_Oct_Scu_Wed_MBIC_Vers1_100-12.5.PNG	Oct	12.50	0.4063337	0.0062472	0.4056698	0.0098039
EGCG_Oct_Scu_Wed_MBIC_Vers2_100-12.5.PNG	Oct	100.00	0.3978898	0.0066341	0.3978682	0.0081961
EGCG_Oct_Scu_Wed_MBIC_Vers2_100-12.5.PNG	Oct	75.00	0.4227679	0.0096638	0.4213976	0.0121961
EGCG_Oct_Scu_Wed_MBIC_Vers2_100-12.5.PNG	Oct	50.00	0.4157612	0.0058397	0.4157113	0.0078824
EGCG_Oct_Scu_Wed_MBIC_Vers2_100-12.5.PNG	Oct	25.00	0.4148669	0.0136717	0.4133976	0.0186667
EGCG_Oct_Scu_Wed_MBIC_Vers2_100-12.5.PNG	Oct	12.50	0.2888640	0.0412259	0.2784956	0.0541176
EGCG_Oct_Scu_Wed_MBIC_Vers3_100-12.5mg.PNG	Oct	100.00	0.4063780	0.0059721	0.4058799	0.0078431
EGCG_Oct_Scu_Wed_MBIC_Vers3_100-12.5mg.PNG	Oct	75.00	0.4264096	0.0070665	0.4246642	0.0078824
EGCG_Oct_Scu_Wed_MBIC_Vers3_100-12.5mg.PNG	Oct	50.00	0.4560956	0.0183135	0.4499191	0.0271373
EGCG_Oct_Scu_Wed_MBIC_Vers3_100-12.5mg.PNG	Oct	25.00	0.4461089	0.0096205	0.4447819	0.0144314
EGCG_Oct_Scu_Wed_MBIC_Vers3_100-12.5mg.PNG	Oct	12.50	0.4249628	0.0110589	0.4220760	0.0180392
EGCG_Oct_Scu_Wed_MBIC_Vers3_100-1.25.PNG	Oct	10.00	0.2380083	0.1097903	0.2078977	0.0263137
EGCG_Oct_Scu_Wed_MBIC_Vers3_100-1.25.PNG	Oct	7.50	0.2195603	0.0152569	0.2157409	0.0123922
EGCG_Oct_Scu_Wed_MBIC_Vers3_100-1.25.PNG	Oct	5.00	0.6116820	0.1810540	0.6153291	0.3060392
EGCG_Oct_Scu_Wed_MBIC_Vers3_100-1.25.PNG	Oct	2.50	0.5290840	0.1918710	0.5001134	0.3143431
EGCG_Oct_Scu_Wed_MBIC_Vers3_100-1.25.PNG	Oct	1.25	0.4941446	0.1473680	0.4704468	0.2121961
EGCG_Oct_Scu_Wed_MBIC_Vers4_100-12.5.PNG	Oct	100.00	0.3894330	0.0183350	0.3877345	0.0288235
EGCG_Oct_Scu_Wed_MBIC_Vers4_100-12.5.PNG	Oct	75.00	0.4136250	0.0140256	0.4113815	0.0206667
EGCG_Oct_Scu_Wed_MBIC_Vers4_100-12.5.PNG	Oct	50.00	0.4285583	0.0119193	0.4264404	0.0190980
EGCG_Oct_Scu_Wed_MBIC_Vers4_100-12.5.PNG	Oct	25.00	0.4338199	0.0154483	0.4301266	0.0151765
EGCG_Oct_Scu_Wed_MBIC_Vers4_100-12.5.PNG	Oct	12.50	0.3234055	0.0158823	0.3191070	0.0240784
EGCG_Oct_Scu_Wed_MBIC_Vers4_1-1.25_Reihe2.PNG	Oct	10.00	0.2319266	0.0106387	0.2309614	0.0156275
EGCG_Oct_Scu_Wed_MBIC_Vers4_1-1.25_Reihe2.PNG	Oct	7.50	0.2278136	0.0122830	0.2235888	0.0139608
EGCG_Oct_Scu_Wed_MBIC_Vers4_1-1.25_Reihe2.PNG	Oct	5.00	0.4190632	0.1517448	0.3720986	0.2267843
EGCG_Oct_Scu_Wed_MBIC_Vers4_1-1.25_Reihe2.PNG	Oct	2.50	0.5432547	0.1960261	0.5391574	0.3537157
EGCG_Oct_Scu_Wed_MBIC_Vers4_1-1.25_Reihe2.PNG	Oct	1.25	0.4767053	0.2202616	0.3610790	0.2670882

image_name	substances	conc	mean	sd	median	IQR
EGCG_Oct_Scu_Wed_MBIC_Vers5_100-12.5_Reihe2.PNG	Oct	100.00	0.3844165	0.0113501	0.3835408	0.0156863
EGCG_Oct_Scu_Wed_MBIC_Vers5_100-12.5_Reihe2.PNG	Oct	75.00	0.3948478	0.0102136	0.3923251	0.0105490
EGCG_Oct_Scu_Wed_MBIC_Vers5_100-12.5_Reihe2.PNG	Oct	50.00	0.4044053	0.0107482	0.4026780	0.0121176
EGCG_Oct_Scu_Wed_MBIC_Vers5_100-12.5_Reihe2.PNG	Oct	25.00	0.3661481	0.0152706	0.3688741	0.0238824
EGCG_Oct_Scu_Wed_MBIC_Vers5_100-12.5_Reihe2.PNG	Oct	12.50	0.2871857	0.0090188	0.2864820	0.0130196
EGCG_Oct_Scu_Wed_MBIC_Vers5_1-1.25_Reihe2.PNG	Oct	10.00	0.2345468	0.0064784	0.2342517	0.0085882
EGCG_Oct_Scu_Wed_MBIC_Vers5_1-1.25_Reihe2.PNG	Oct	7.50	0.2546027	0.0089253	0.2538595	0.0117647
EGCG_Oct_Scu_Wed_MBIC_Vers5_1-1.25_Reihe2.PNG	Oct	5.00	0.3993616	0.1089206	0.3720948	0.1423137
EGCG_Oct_Scu_Wed_MBIC_Vers5_1-1.25_Reihe2.PNG	Oct	2.50	0.4550451	0.1210486	0.4265654	0.1566471
EGCG_Oct_Scu_Wed_MBIC_Vers5_1-1.25_Reihe2.PNG	Oct	1.25	0.3990506	0.0787880	0.3805262	0.1011569
EGCG_Oct_Scu_Wed_MBIC_Vers1_100-12.5.PNG	Scu	100.00	0.5639779	0.0123956	0.5613953	0.0146275
EGCG_Oct_Scu_Wed_MBIC_Vers1_100-12.5.PNG	Scu	75.00	0.5717727	0.0174019	0.5662188	0.0173725
EGCG_Oct_Scu_Wed_MBIC_Vers1_100-12.5.PNG	Scu	50.00	0.5287640	0.0076551	0.5271600	0.0076078
EGCG_Oct_Scu_Wed_MBIC_Vers1_100-12.5.PNG	Scu	25.00	0.5078002	0.0116862	0.5060619	0.0081569
EGCG_Oct_Scu_Wed_MBIC_Vers1_100-12.5.PNG	Scu	12.50	0.4170253	0.0107236	0.4160619	0.0142353
EGCG_Oct_Scu_Wed_MBIC_Vers2_100-12.5.PNG	Scu	100.00	0.4346263	0.0141343	0.4322603	0.0125098
EGCG_Oct_Scu_Wed_MBIC_Vers2_100-12.5.PNG	Scu	75.00	0.4046721	0.0102891	0.4057505	0.0164314
EGCG_Oct_Scu_Wed_MBIC_Vers2_100-12.5.PNG	Scu	50.00	0.4158521	0.0152521	0.4132799	0.0128627
EGCG_Oct_Scu_Wed_MBIC_Vers2_100-12.5.PNG	Scu	25.00	0.2776878	0.0066306	0.2763388	0.0086275
EGCG_Oct_Scu_Wed_MBIC_Vers2_100-12.5.PNG	Scu	12.50	0.2534865	0.0198735	0.2473976	0.0286961
EGCG_Oct_Scu_Wed_MBIC_Vers3_100-12.5mg.PNG	Scu	100.00	0.5043260	0.0143992	0.5005466	0.0216078
EGCG_Oct_Scu_Wed_MBIC_Vers3_100-12.5mg.PNG	Scu	75.00	0.4891250	0.0127986	0.4847819	0.0172549
EGCG_Oct_Scu_Wed_MBIC_Vers3_100-12.5mg.PNG	Scu	50.00	0.4867272	0.0131345	0.4839976	0.0184314
EGCG_Oct_Scu_Wed_MBIC_Vers3_100-12.5mg.PNG	Scu	25.00	0.4454917	0.0101087	0.4444290	0.0149412
EGCG_Oct_Scu_Wed_MBIC_Vers3_100-12.5mg.PNG	Scu	12.50	0.3640900	0.0090467	0.3619976	0.0121176
EGCG_Oct_Scu_Wed_MBIC_Vers3_100-1.25.PNG	Scu	10.00	0.4661500	0.1798643	0.4205644	0.2938922

image_name	substances	conc	mean	sd	median	IQR
EGCG_Oct_Scu_Wed_MBIC_Vers3_100-1.25.PNG	Scu	7.50	0.4688803	0.1398391	0.4467213	0.2089706
EGCG_Oct_Scu_Wed_MBIC_Vers3_100-1.25.PNG	Scu	5.00	0.4976835	0.1679433	0.4668781	0.2697059
EGCG_Oct_Scu_Wed_MBIC_Vers3_100-1.25.PNG	Scu	2.50	0.5022492	0.1678046	0.4743879	0.2477843
EGCG_Oct_Scu_Wed_MBIC_Vers3_100-1.25.PNG	Scu	1.25	0.3209437	0.0818313	0.2946820	0.0745098
EGCG_Oct_Scu_Wed_MBIC_Vers4_100-12.5.PNG	Scu	100.00	0.3347714	0.0254153	0.3278129	0.0203039
EGCG_Oct_Scu_Wed_MBIC_Vers4_100-12.5.PNG	Scu	75.00	0.3374664	0.0141284	0.3352247	0.0148627
EGCG_Oct_Scu_Wed_MBIC_Vers4_100-12.5.PNG	Scu	50.00	0.2870781	0.0094743	0.2864796	0.0128627
EGCG_Oct_Scu_Wed_MBIC_Vers4_100-12.5.PNG	Scu	25.00	0.2781722	0.0173404	0.2740090	0.0229804
EGCG_Oct_Scu_Wed_MBIC_Vers4_100-12.5.PNG	Scu	12.50	0.2478894	0.0192868	0.2427541	0.0294510
EGCG_Oct_Scu_Wed_MBIC_Vers4_1-1.25_Reihe2.PNG	Scu	10.00	0.3985809	0.1075805	0.3751378	0.1532745
EGCG_Oct_Scu_Wed_MBIC_Vers4_1-1.25_Reihe2.PNG	Scu	7.50	0.4621654	0.1193860	0.4373731	0.1747745
EGCG_Oct_Scu_Wed_MBIC_Vers4_1-1.25_Reihe2.PNG	Scu	5.00	0.3142523	0.0930328	0.2826868	0.0634804
EGCG_Oct_Scu_Wed_MBIC_Vers4_1-1.25_Reihe2.PNG	Scu	2.50	0.3384779	0.1391662	0.2861378	0.1019510
EGCG_Oct_Scu_Wed_MBIC_Vers4_1-1.25_Reihe2.PNG	Scu	1.25	0.4461411	0.1275536	0.4047653	0.1252353
EGCG_Oct_Scu_Wed_MBIC_Vers5_100-12.5_Reihe2.PNG	Scu	100.00	0.4525197	0.0224090	0.4464035	0.0195686
EGCG_Oct_Scu_Wed_MBIC_Vers5_100-12.5_Reihe2.PNG	Scu	75.00	0.4701624	0.0192142	0.4632663	0.0153725
EGCG_Oct_Scu_Wed_MBIC_Vers5_100-12.5_Reihe2.PNG	Scu	50.00	0.4316601	0.0075395	0.4311486	0.0100784
EGCG_Oct_Scu_Wed_MBIC_Vers5_100-12.5_Reihe2.PNG	Scu	25.00	0.3075309	0.0099806	0.3056584	0.0125882
EGCG_Oct_Scu_Wed_MBIC_Vers5_100-12.5_Reihe2.PNG	Scu	12.50	0.2916510	0.0132257	0.2898937	0.0196078
EGCG_Oct_Scu_Wed_MBIC_Vers5_1-1.25_Reihe2.PNG	Scu	10.00	0.3967695	0.0513011	0.3883693	0.0534118
EGCG_Oct_Scu_Wed_MBIC_Vers5_1-1.25_Reihe2.PNG	Scu	7.50	0.4108199	0.0586124	0.3987419	0.0641961
EGCG_Oct_Scu_Wed_MBIC_Vers5_1-1.25_Reihe2.PNG	Scu	5.00	0.4259712	0.0535102	0.4154281	0.0662549
EGCG_Oct_Scu_Wed_MBIC_Vers5_1-1.25_Reihe2.PNG	Scu	2.50	0.4259250	0.0535254	0.4153105	0.0668627
EGCG_Oct_Scu_Wed_MBIC_Vers5_1-1.25_Reihe2.PNG	Scu	1.25	0.3954183	0.0575343	0.3831144	0.0700490
EGCG_Oct_Scu_Wed_MBIC_Vers1_100-12.5.PNG	Wed	100.00	0.5673478	0.0077444	0.5654737	0.0109412
EGCG_Oct_Scu_Wed_MBIC_Vers1_100-12.5.PNG	Wed	75.00	0.5467288	0.0040966	0.5462580	0.0050980

image_name	substances	conc	mean	sd	median	IQR
EGCG_Oct_Scu_Wed_MBIC_Vers1_100-12.5.PNG	Wed	50.00	0.5097422	0.0039944	0.5093561	0.0066275
EGCG_Oct_Scu_Wed_MBIC_Vers1_100-12.5.PNG	Wed	25.00	0.4813353	0.0060679	0.4817090	0.0077255
EGCG_Oct_Scu_Wed_MBIC_Vers1_100-12.5.PNG	Wed	12.50	0.4021570	0.0108598	0.4002972	0.0119216
EGCG_Oct_Scu_Wed_MBIC_Vers2_100-12.5.PNG	Wed	100.00	0.3595723	0.0096162	0.3586525	0.0137255
EGCG_Oct_Scu_Wed_MBIC_Vers2_100-12.5.PNG	Wed	75.00	0.3193213	0.0166403	0.3193976	0.0238431
EGCG_Oct_Scu_Wed_MBIC_Vers2_100-12.5.PNG	Wed	50.00	0.2799633	0.0211527	0.2785348	0.0358039
EGCG_Oct_Scu_Wed_MBIC_Vers2_100-12.5.PNG	Wed	25.00	0.2641529	0.0190144	0.2593976	0.0305098
EGCG_Oct_Scu_Wed_MBIC_Vers2_100-12.5.PNG	Wed	12.50	0.2460274	0.0201475	0.2448878	0.0188235
EGCG_Oct_Scu_Wed_MBIC_Vers3_100-12.5mg.PNG	Wed	100.00	0.4756053	0.0158033	0.4716054	0.0230980
EGCG_Oct_Scu_Wed_MBIC_Vers3_100-12.5mg.PNG	Wed	75.00	0.4650528	0.0123747	0.4618407	0.0162353
EGCG_Oct_Scu_Wed_MBIC_Vers3_100-12.5mg.PNG	Wed	50.00	0.4434712	0.0120657	0.4412133	0.0196471
EGCG_Oct_Scu_Wed_MBIC_Vers3_100-12.5mg.PNG	Wed	25.00	0.4016651	0.0109458	0.3986642	0.0129020
EGCG_Oct_Scu_Wed_MBIC_Vers3_100-12.5mg.PNG	Wed	12.50	0.3815744	0.0088694	0.3795662	0.0127059
EGCG_Oct_Scu_Wed_MBIC_Vers3_100-1.25.PNG	Wed	10.00	0.3949599	0.1169327	0.3650938	0.1495588
EGCG_Oct_Scu_Wed_MBIC_Vers3_100-1.25.PNG	Wed	7.50	0.4004369	0.1211266	0.3675448	0.1455588
EGCG_Oct_Scu_Wed_MBIC_Vers3_100-1.25.PNG	Wed	5.00	0.4307116	0.1084036	0.4111134	0.1590588
EGCG_Oct_Scu_Wed_MBIC_Vers3_100-1.25.PNG	Wed	2.50	0.4050177	0.1094486	0.3800938	0.1431373
EGCG_Oct_Scu_Wed_MBIC_Vers3_100-1.25.PNG	Wed	1.25	0.2760812	0.0648839	0.2553095	0.0566275
EGCG_Oct_Scu_Wed_MBIC_Vers4_100-12.5.PNG	Wed	100.00	0.3787334	0.0134333	0.3790678	0.0146275
EGCG_Oct_Scu_Wed_MBIC_Vers4_100-12.5.PNG	Wed	75.00	0.3371710	0.0157526	0.3324404	0.0191176
EGCG_Oct_Scu_Wed_MBIC_Vers4_100-12.5.PNG	Wed	50.00	0.2775330	0.0412211	0.2919306	0.0552451
EGCG_Oct_Scu_Wed_MBIC_Vers4_100-12.5.PNG	Wed	25.00	0.2215414	0.0337576	0.2255776	0.0527451
EGCG_Oct_Scu_Wed_MBIC_Vers4_100-12.5.PNG	Wed	12.50	0.2209294	0.0177390	0.2183619	0.0265882
EGCG_Oct_Scu_Wed_MBIC_Vers4_1-1.25_Reihe2.PNG	Wed	10.00	0.2898531	0.0982504	0.2716084	0.1251373
EGCG_Oct_Scu_Wed_MBIC_Vers4_1-1.25_Reihe2.PNG	Wed	7.50	0.2980293	0.1111793	0.2880986	0.1306078
EGCG_Oct_Scu_Wed_MBIC_Vers4_1-1.25_Reihe2.PNG	Wed	5.00	0.3157078	0.1191843	0.2665300	0.0704118

image_name	substances	conc	mean	sd	median	IQR
EGCG_Oct_Scu_Wed_MBIC_Vers4_1-1.25_Reihe2.PNG	Wed	2.50	0.3427252	0.1660247	0.2745300	0.1096275
EGCG_Oct_Scu_Wed_MBIC_Vers4_1-1.25_Reihe2.PNG	Wed	1.25	0.3737127	0.1853266	0.2859417	0.1061471
EGCG_Oct_Scu_Wed_MBIC_Vers5_100-12.5_Reihe2.PNG	Wed	100.00	0.4458675	0.0063299	0.4466780	0.0086275
EGCG_Oct_Scu_Wed_MBIC_Vers5_100-12.5_Reihe2.PNG	Wed	75.00	0.3859755	0.0112504	0.3866780	0.0141961
EGCG_Oct_Scu_Wed_MBIC_Vers5_100-12.5_Reihe2.PNG	Wed	50.00	0.3732166	0.0148490	0.3715800	0.0176863
EGCG_Oct_Scu_Wed_MBIC_Vers5_100-12.5_Reihe2.PNG	Wed	25.00	0.3176853	0.0222895	0.3163643	0.0328529
EGCG_Oct_Scu_Wed_MBIC_Vers5_100-12.5_Reihe2.PNG	Wed	12.50	0.2840022	0.0130917	0.2820506	0.0185098
EGCG_Oct_Scu_Wed_MBIC_Vers5_1-1.25_Reihe2.PNG	Wed	10.00	0.3418226	0.0554953	0.3329183	0.0626275
EGCG_Oct_Scu_Wed_MBIC_Vers5_1-1.25_Reihe2.PNG	Wed	7.50	0.3631740	0.0561765	0.3516634	0.0619412
EGCG_Oct_Scu_Wed_MBIC_Vers5_1-1.25_Reihe2.PNG	Wed	5.00	0.3859070	0.0628019	0.3762125	0.0707843
EGCG_Oct_Scu_Wed_MBIC_Vers5_1-1.25_Reihe2.PNG	Wed	2.50	0.4064510	0.0661580	0.3959772	0.0752059
EGCG_Oct_Scu_Wed_MBIC_Vers5_1-1.25_Reihe2.PNG	Wed	1.25	0.3618470	0.0666436	0.3599576	0.0963039

References

Rödiger, Stefan, Thomas Friedrichsmeier, Prasenjit Kapat, and Meik Michalke. 2012. "RKWard: a comprehensive graphical user interface and integrated development environment for statistical analysis with R." *Journal of Statistical Software* 49 (9): 1–34. https://doi.org/10.18637/jss.v049.i09.

Schneider, Jens, Romano Weiss, Madeleine Ruhe, Tobias Jung, Dirk Roggenbuck, Ralf Stohwasser, Peter Schierack, and Stefan Rödiger. 2019. "Open source bioimage informatics tools for the analysis of DNA damage and associated biomarkers." *Journal of Laboratory and Precision Medicine* 4 (0): 1–27. https://doi.org/10.21037/jlpm.2019.04.05.
