# Supplementary material for: Natural phenolic compounds as biofilm inhibitors of multidrug-resistant Escherichia coli – the role of similar biological processes despite structural diversity
Source: Front Microbiol. 2023 Sep 4;14:1232039. doi: 10.3389/fmicb.2023.1232039 (PMC10507321; doi:10.3389/fmicb.2023.1232039)

SUPPLEMENTARY FIGURE 1: Scanning electron micrographs of DMSO (A), EGCG (B), octyl gallate (C), scutellarein (D) and wedelolactone (E) treated macro colonies of E. coli PBIO729 at 2500x magnification. Macro colonies were grown on a 0.2 µm polycarbonate filter on span agar for 48 h at 28 °C. Scale bars = 2 µm.

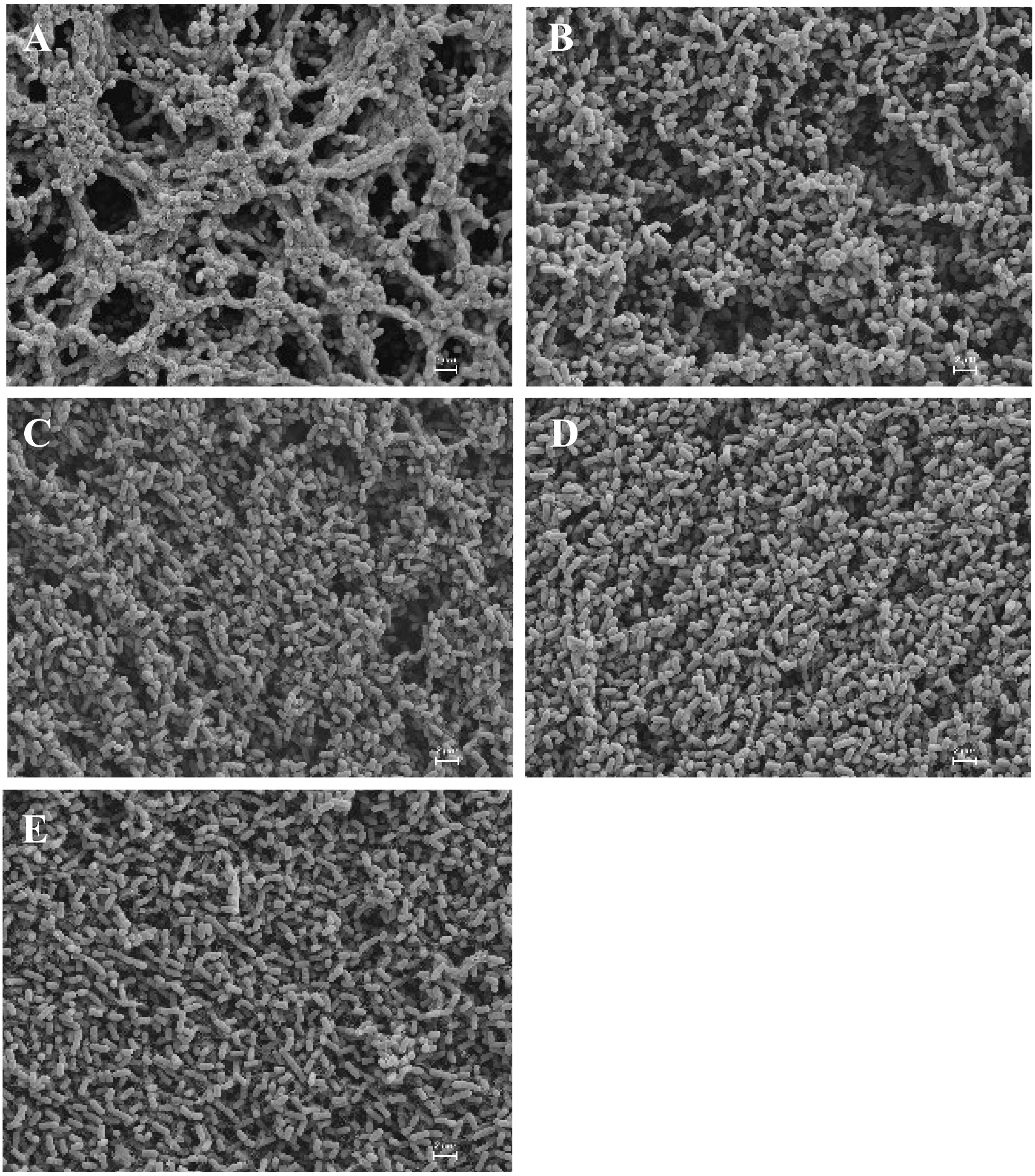

Supplement: Supplementary file 6 [file Image_1.pdf]
