# Supplementary material for: Natural phenolic compounds as biofilm inhibitors of multidrug-resistant Escherichia coli – the role of similar biological processes despite structural diversity
Source: Front Microbiol. 2023 Sep 4;14:1232039. doi: 10.3389/fmicb.2023.1232039 (PMC10507321; doi:10.3389/fmicb.2023.1232039)

SUPPLEMENTARY FIGURE 2. Plot of principle component analysis of RNAseq data of treated biofilms with the inhibitors epigallocatechin gallate (EGCG), octyl gallate (Oct), scutellarein (Scu) and wedelolactone (Wed) compared with the control group DMSO. Displayed are all biological replicates.

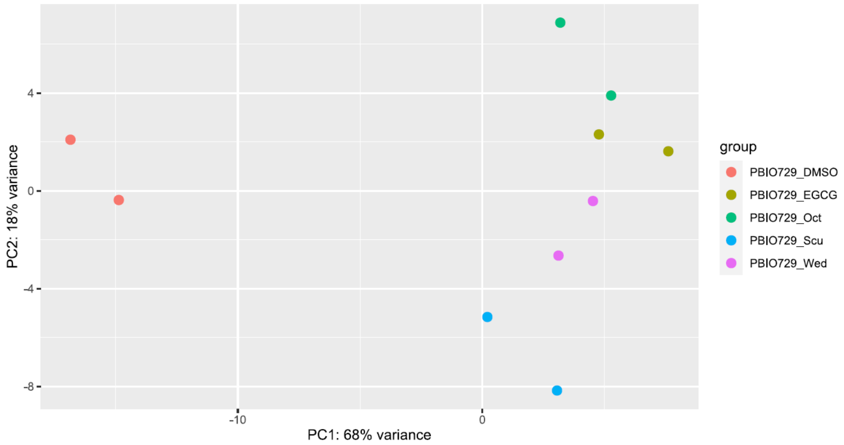

Supplement: Supplementary file 7 [file Image_2.pdf]
