# Supplementary material for: Natural phenolic compounds as biofilm inhibitors of multidrug-resistant Escherichia coli – the role of similar biological processes despite structural diversity
Source: Front Microbiol. 2023 Sep 4;14:1232039. doi: 10.3389/fmicb.2023.1232039 (PMC10507321; doi:10.3389/fmicb.2023.1232039)

SUPPLEMENTARY FIGURE 3. Enrichment of Gene Ontology categories for the differential expressed genes after treatment of *E. coli* biofilm formation with EGCG, octyl gallate, scutellarein and wedelolactone. The figure displays the most significant GO terms in EC958 after analysis with fisher exact test, p-value = 0.1 and Benjamini-Hochberg-correction.

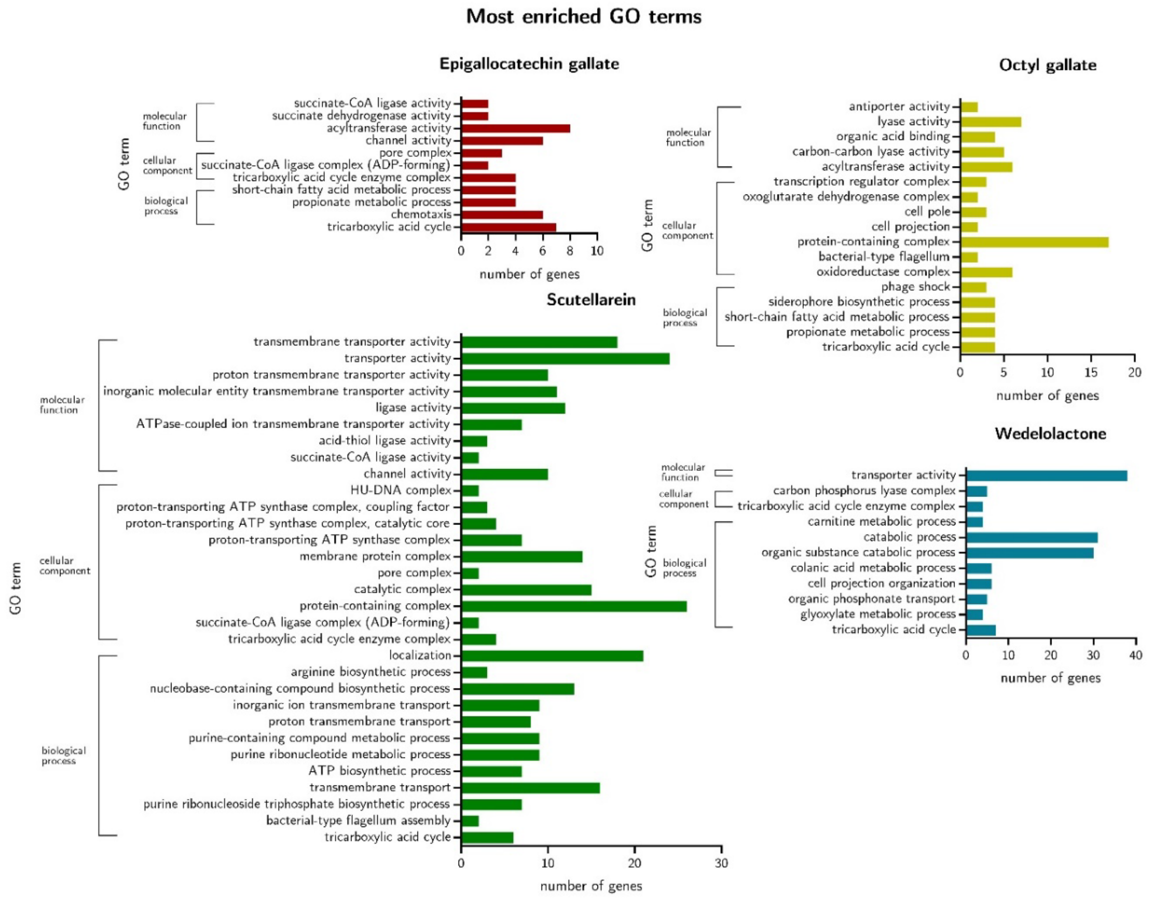

Supplement: Supplementary file 8 [file Image_3.pdf]
